# The nucleic acid chaperone activity of the HIV-1 Gag polyprotein is boosted by its cellular partner RPL7: a kinetic study

**DOI:** 10.1093/nar/gkaa659

**Published:** 2020-08-14

**Authors:** Hassan Karnib, Muhammad F Nadeem, Nicolas Humbert, Kamal K Sharma, Natalia Grytsyk, Carine Tisné, Emmanuel Boutant, Thiebault Lequeu, Eleonore Réal, Christian Boudier, Hugues de Rocquigny, Yves Mély

**Affiliations:** Laboratory of Bioimaging and Pathologies (LBP), UMR 7021, Faculty of pharmacy, University of Strasbourg, 67400 Illkirch, France; Laboratory of Bioimaging and Pathologies (LBP), UMR 7021, Faculty of pharmacy, University of Strasbourg, 67400 Illkirch, France; Laboratory of Bioimaging and Pathologies (LBP), UMR 7021, Faculty of pharmacy, University of Strasbourg, 67400 Illkirch, France; Laboratory of Bioimaging and Pathologies (LBP), UMR 7021, Faculty of pharmacy, University of Strasbourg, 67400 Illkirch, France; Laboratory of Bioimaging and Pathologies (LBP), UMR 7021, Faculty of pharmacy, University of Strasbourg, 67400 Illkirch, France; Expression génétique microbienne, UMR 8261, CNRS, Université de Paris, Institut de biologie physico-chimique, 13 rue Pierre et Marie Curie, 75005 Paris, France; Laboratory of Bioimaging and Pathologies (LBP), UMR 7021, Faculty of pharmacy, University of Strasbourg, 67400 Illkirch, France; Laboratory of Bioimaging and Pathologies (LBP), UMR 7021, Faculty of pharmacy, University of Strasbourg, 67400 Illkirch, France; Laboratory of Bioimaging and Pathologies (LBP), UMR 7021, Faculty of pharmacy, University of Strasbourg, 67400 Illkirch, France; Laboratory of Bioimaging and Pathologies (LBP), UMR 7021, Faculty of pharmacy, University of Strasbourg, 67400 Illkirch, France; Inserm – U1259 Morphogenesis and Antigenicity of HIV and Hepatitis Viruses (MAVIVH), 10 boulevard Tonnellé, BP 3223, 37032 Tours Cedex 1, France; Laboratory of Bioimaging and Pathologies (LBP), UMR 7021, Faculty of pharmacy, University of Strasbourg, 67400 Illkirch, France

## Abstract

The HIV-1 Gag protein playing a key role in HIV-1 viral assembly has recently been shown to interact through its nucleocapsid domain with the ribosomal protein L7 (RPL7) that acts as a cellular co-factor promoting Gag's nucleic acid (NA) chaperone activity. To further understand how the two proteins act together, we examined their mechanism individually and in concert to promote the annealing between dTAR, the DNA version of the viral transactivation element and its complementary cTAR sequence, taken as model HIV-1 sequences. Gag alone or complexed with RPL7 was found to act as a NA chaperone that destabilizes cTAR stem-loop and promotes its annealing with dTAR through the stem ends via a two-step pathway. In contrast, RPL7 alone acts as a NA annealer that through its NA aggregating properties promotes cTAR/dTAR annealing via two parallel pathways. Remarkably, in contrast to the isolated proteins, their complex promoted efficiently the annealing of cTAR with highly stable dTAR mutants. This was confirmed by the RPL7-promoted boost of the physiologically relevant Gag-chaperoned annealing of (+)PBS RNA to the highly stable tRNA^Lys^_3_ primer, favoring the notion that Gag recruits RPL7 to overcome major roadblocks in viral assembly.

## INTRODUCTION

In human immunodeficiency type 1 (HIV-1) infected cells, the integrated viral DNA is transcribed by the host cell machinery generating the full-length viral RNA (FL RNA). Once exported to the cytoplasm, the FL RNA can be recruited by active ribosomes to direct the synthesis of Gag and GagPol precursors. The 55 kDa Gag polyprotein precursors (herein after named as ‘Gag’) play a critical role in orchestrating the formation of new virus particles, by selecting the FL RNA among the bulk of cellular RNAs, and promoting its dimerization and encapsidation into the new viral particles (1–4). Both the FL RNA that will be used as genomic RNA (gRNA) in newly assembled viruses and the plasma membrane of the host cell can be perceived as platforms used to promote Gag multimerization along viral assembly. The Gag protein plays also a key role in promoting the annealing of the 3′ terminal 18 nucleotides (nts) of the tRNA^Lys^_3_ primer to the primer binding site (PBS) of the genomic RNA (for a review, see (5,6)) during production of new viruses. The Gag protein consists of the matrix (MA), capsid (CA), nucleocapsid (NC) and p6 domains (2,7) that all contribute to the overall activity of Gag. The MA domain targets Gag to the plasma membrane during virus assembly and also binds RNA (8–11). The CA domain facilitates Gag multimerization through CA–CA interaction (12,13). The NC domain is required for genomic RNA selection, dimerization and packaging (14,15) as well as primer placement (16–18). The p6 domain facilitates the budding of viral particles through recruitment of TSG101 and ALIX of the ESCRT family (2,19–21).

Proteolytic maturation of Gag leads to its various components in their free form including the 55 amino acids protein NCp7 that can associate with nucleic acids (NAs) to form a variety of complexes (2,22). Through these interactions, NCp7 can structurally rearrange NAs into their most thermodynamically stable conformation (23–26). This NA chaperone (NAC) activity of NCp7 and its binding specificity mainly rely on its two highly conserved zinc fingers, connected by a basic peptide. A hydrophobic plateau forms at the top of the folded fingers, conferring to the protein the capacity to dynamically bind and destabilize its NA targets (27–32). In addition, the numerous basic residues in the unfolded regions of the protein account for its annealing (33) and aggregating properties (34). HIV-1 NCp7 crucially contributes to viral DNA synthesis during reverse transcription by promoting its initiation as well as its two obligatory strand transfers (35–39).

The full-length Gag protein can associate tightly with RNA and DNA via its NC domain and possibly its MA domain (8,40–41). Its affinity for DNA was reported to be up to ∼10-fold higher than that of NCp7, depending on the experimental conditions and the oligonucleotide length (42–45). Gag also exhibits NAC activity that is thought to be instrumental in its interaction with FL RNA and the recruitment of the tRNA^Lys^_3_ primer during viral assembly. The NAC activity of Gag was inferred *in vitro* on a reconstituted minus strand transfer reaction (43) and both *in vitro* and *in vivo* for tRNA^Lys^_3_/PBS annealing (17,46), but its mechanism is still ill-defined. Gag's NAC activity is mainly supported by its NC domain, since only partially processed Gag proteins and Gag mutants containing the NC domain show NAC activity (43). Nevertheless, the NAC activity of Gag is lower than that of NCp7 (17–18,42–43), suggesting that Gag would benefit from co-factor(s) to improve its NAC activity (47). Inositol hexakisphosphate (IP6) and inositol phosphate-containing lipids were identified as potential cofactors that through their binding to the MA domain promote Gag's NAC activity on the tRNA^Lys^_3_/PBS system *in vitro* (10). The RNA helicase A was also reported to favor the Gag-promoted tRNA^Lys^_3_/PBS annealing, likely by promoting a viral RNA conformation susceptible to annealing (47). Moreover, we recently reported that the human ribosomal protein L7 (RPL7), that is part of the large ribosomal subunit, can interact with the NC domain of Gag *in cellulo* and *in vitro* (48) and stimulate the Gag-promoted annealing of the well described system made of dTAR, the DNA version of the Trans Active Response element and its complementary cTAR strand (49–52).

In this work, we thoroughly investigated by a combination of fluorescence-based methods the mechanism by which Gag, RPL7 and the Gag–RPL7 complex promote the annealing of cTAR with dTAR derivatives. We evidenced that Gag but not RPL7 can destabilize the cTAR stem-loop. Moreover, we found that both proteins either isolated or in complex promote the annealing reaction through mechanisms involving one or two pathways. Each pathway involves the fast formation of an intermediate complex that is subsequently converted into the final extended duplex (ED). In their complex, RPL7 was observed to activate Gag's NAC properties. As a result, while Gag mainly promotes the annealing of moderately stable stem-loops, the complex can promote the annealing of much more stable sequences. This conclusion was further illustrated by the efficient promotion by the Gag–RPL7 complex of the annealing of the stable tRNA^Lys^_3_ sequence to (+)PBS RNA. Altogether, our results favor the notion that Gag recruits RPL7 to boost its NAC activity during the late phase of HIV-1 replication.

## MATERIALS AND METHODS

### Oligonucleotides

The unlabeled and labeled cTAR and dTAR derivatives and the 18-oligonucleotides (+)PBS RNA were synthesized by IBA GmbH Nucleic Acids product Supply (Gottingen, Germany). For singly labeled oligonucleotides, the 5′ terminus was labelled with 5(6)-carboxytetramethylrhodamine (TMR) or 5(and 6)-carboxyfluorescein (Fl). In the case of the doubly labelled species, the 5′ terminus was labeled with ethyl 2-[3-(ethylamino)-6-ethylimino-2,7-dimethylxanthen-9-yl]benzoate hydrochloride (Rh6G) via an amino-linker with a six carbon spacer arm, while the 3′ terminus was labelled with 4-(4′-dimethylaminophenylazo) benzoic acid (Dabcyl) using a special solid support with the dye already attached. Oligonucleotides were purified by the manufacturer by reverse-phase HPLC and polyacrylamide gel electrophoresis. The oligonucleotides concentration was calculated from the absorbance of their solutions at 260 nm using a molar extinction coefficient of 5.56 × 10^5^ M^−1^.cm^−1^, 5.775 × 10^5^ M^−1^.cm^−1^, 5.15 × 10^5^ M^−1^.cm^−1^ and 2.25 × 10^5^ M^−1^.cm^−1^ for TMR-5′-cTAR, Rh6G-5′-cTAR-3′-Dabcyl, dTAR and its derivatives and Rh6G-5′-(+)PBS RNA-3′-Dabcyl, respectively. The human recombinant tRNA^Lys^_3_ was expressed and purified as previously described (53).

### Preparation of NC(1–55)

**Figure 1.**
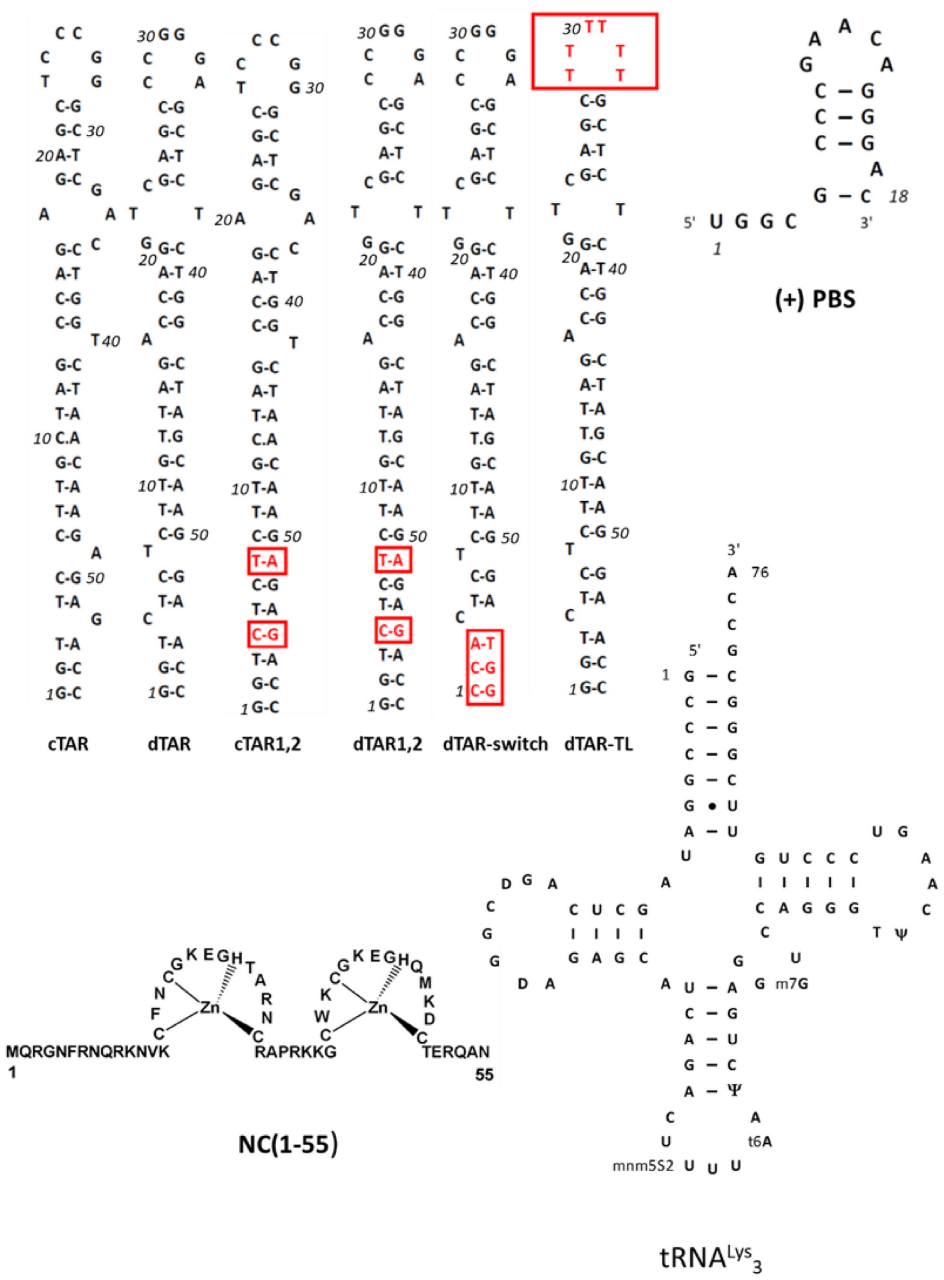
Sequence of the oligonucleotides and the NC(1–55) protein used in this study. The mutations of the cTAR and dTAR mutants are highlighted in red. The base modifications of the recombinant tRNA^Lys^_3_ used in this study are as in (53).

The NC(1–55) peptide that corresponds to the sequence of the mature nucleocapsid protein NCp7 (Figure 1) was synthesized and purified, as previously described (54). Its solution concentration was determined from the absorbance at 280 nm, using a molar extinction coefficient of 5700 M^−1^.cm^−1^. The zinc-bound form of the peptide was freshly prepared by adding a 2.5-fold molar excess of zinc sulphate, as described (55). All experiments were performed in 50 mM Tris–HCl (pH 7.4), 150 mM NaCl, 1 mM MgCl_2_ and 1 mM dithiothreitol (DTT). The rather high salt concentrations mimicking the physiological salt concentrations were selected to limit the non-specific binding and thus, the NA aggregating properties of both Gag and RPL7 proteins.

### Preparation of HIV-1 Gag and human ribosomal protein L7 (RPL7)

Human RPL7 was produced as already described (56). TEV-cleavable HIV-1 Gag was produced as already described (44) with a few modifications. Briefly, after loading the cleared bacterial lysate onto a 5-ml His-Trap HP column, the Gag-TEV-His-containing fractions were pooled and desalted within PD-10 columns, then digested with 1.2 kU of TEV protease, overnight at 4°C. The resulting product was loaded on 1 ml HisTrap column to remove both the cleaved His-tag and the tagged enzyme. The unbound material was collected, concentrated and applied on a High Load 16/60 Superdex 200 size exclusion column (GE Healthcare). Finally, the Gag-containing fractions were pooled, concentrated to 1–2 mg/ml, snap frozen in liquid nitrogen and stored at −80°C. The purity of Gag (Supplementary Figure S1) and RPL7 was checked by polyacrylamide gel electrophoresis. The ratios of the absorbance at 260 nm to that at 280 nm were close to 0.6, indicating that both proteins were marginally contaminated by nucleic acids (57). Their concentration was calculated from their absorbance at 280 nm using molar extinction coefficients of 62 050 L.mol^−1^.cm^−1^ and 27 390 L.mol^−1^.cm^−1^ for Gag and RPL7, respectively.

### Fluorescence correlation spectroscopy measurements and data analysis

Fluorescence correlation spectroscopy (FCS) measurements were performed on a two-photon microscope based on an Olympus IX70 inverted microscope, as described previously (58). Two-photon excitation at 850 nm is provided by an InSight DeepSee laser source (Spectra Physics, USA). The experiments were performed in an 8 well lab-Tek II coverglass system, using a 200-μl volume per well. The focal spot was set about 20 μm above the coverslip. Proteins were added to the labelled cTAR sequences at different molar ratios. To avoid aggregation of the reagents, possibly induced by high local concentrations, the reaction media were made by mixing two identical volumes of protein and oligonucleotide. Assuming that the fluorescent molecules diffuse freely in a Gaussian excitation volume, the normalized autocorrelation function G($\tau$), calculated on-line from the fluorescence fluctuations with a hardware correlator (ALV5000, ALV GmbH, Germany) was fitted according to (59):

(1) $$\begin{equation*}G\left( \tau \right) = \frac{1}{N}{\left. {\left( {1 + \frac{\tau }{{\tau _d}}} \right.} \right)^{ - 1}}{\left. {\left( {1 + \frac{1}{{{S^2}}}\frac{\tau }{{\tau _d}}} \right.} \right)^{ - 1/2}}\end{equation*}$$

where, τ*_d_* is the diffusion time, *N* is the mean number of diffusing species within the excitation volume and *S* is the ratio between the axial and lateral radii of the excitation volume. The point spread function of the set-up was determined from a z-scan on one fluorescent bead (20 nm in diameter). The measured lateral and axial resolutions were respectively, 0.3 and 1 μm. The data were typically recorded during 10 min. Fifty to sixty autocorrelation curves were recorded for each sample. When spikes of high fluorescence intensity, most likely associated to aggregates, were observed in the fluorescence fluctuation profiles, the corresponding autocorrelation curves were discarded. In all conditions, the average autocorrelation curve calculated from the sum of the autocorrelation curves could be adequately fitted by a single population model.

### Fluorescence measurements

Emission spectra, fluorescence anisotropy and kinetic traces were recorded with a FluoroMax 3 or a FluoroLog spectrofluorimeter (Horiba, Jobin Yvon) equipped with a temperature-controlled cell compartment. All fluorescence intensities were corrected for buffer emission and lamp fluctuations as well as for wavelength-dependent response of the optics and detectors. Binding titrations were performed by addition of increasing concentrations of Gag, RPL7 and Gag–RPL7 to 10 or 30 nM Fl-5′-cTAR and monitoring the increase in the fluorescence anisotropy *S* resulting from the formation of the protein/ Fl-5′-cTAR complex. Excitation wavelength was 480 nm and emission wavelength was 520 nm. The anisotropy binding curves were fitted using:

(2) $$\begin{eqnarray*}S &=& \frac{{{S_0} + Y\frac{{{R_a}\left( {{S_t} - {S_0}} \right)}}{n}}}{{1 + Y\frac{{\left( {{R_a} - 1} \right)}}{n}}}{\rm{\;\;with\;}} \nonumber \\ Y &=& {\rm{\;}}\left( {\frac{{1 + ({{\rm{P}}_t} + {\rm{n}}{{\rm{N}}_t})K - \sqrt {\left( {1 + ({{\rm{P}}_t} + {\rm{n}}{{\rm{N}}_t}} \right)K{)^2} - 4{{\rm{P}}_t}n{{\rm{N}}_t}{K^2}} }}{{2K{{\rm{N}}_t}}}} \right)\end{eqnarray*}$$

Where *S_0_* and *S_t_* correspond to the initial and final anisotropy values respectively, *R_a_* the ratio of the fluorescence quantum yield (QY) of the free and bound species, *n* designates the number of binding sites, P*_t_* and N*_t_* correspond to the concentration of protein and Fl-5′-cTAR, respectively. *K* is the equilibrium association constant, which is then converted into a dissociation constant by: *K*_d_ = 1/*K*.

Kinetic measurements were performed in pseudo first-order conditions by reacting unlabeled wild-type or mutant dTAR at a concentration at least 10-fold higher than that of the labeled complementary cTAR sequence. The protein to oligonucleotide molar ratio was 1:1 or 2:1 for Gag, RPL7 and Gag–RPL7 and varying from 1:1 to 4:1 for NC(1–55). The fluorescence emission of Rh6G was monitored at 555 nm with excitation at 520 nm. All reported concentrations correspond to those after mixing. To avoid aggregation possibly resulting from high local concentrations, the reaction was initiated by mixing 400 μl of a solution of dTAR and protein(s) with the same volume of a solution of doubly-labelled cTAR and protein(s). The fitting of the kinetic curves was carried out with the OriginTM software (V9.1) based on non-linear, least-square methods and the Levenberg–Marquardt algorithm. Binding and kinetic experiments were performed in 50 mM Tris–HCl (pH 7.4), 150 mM NaCl, 1 mM MgCl_2_ and 1 mM DTT at 20°C. The temperature dependence of the annealing kinetics was carried out by reacting 10 nM doubly labeled cTAR with 100 nM non-labeled dTAR derivatives at various temperatures, in the presence of Gag, human RPL7 or Gag–RPL7 complex added at a protein to oligonucleotide ratio of 1:1.

## RESULTS

### Determining non-aggregating conditions by fluorescence correlation spectroscopy

In order to characterize by fluorescence-based approaches the NAC properties of Gag, RPL7 and Gag–RPL7 complex, we first determined the experimental conditions under which the studied protein/DNA systems do not significantly aggregate. To this end, we used FCS to investigate the effect of increasing concentrations of Gag, RPL7 or their complex to a fixed amount of TMR- 5′-cTAR (Figure 1).

In this technique, fluorescence intensity fluctuations are measured with time in the very small volume (∼0.2 fl) provided by a two-photon excitation. These fluctuations are mainly governed by the diffusion of the fluorescent species throughout this excitation volume. They are then converted into a correlation function to obtain parameters such as the average number of fluorescent species within this volume and their diffusion constant. Aggregation is thus expected to decrease the number of fluorescent species.

**Figure 2.**
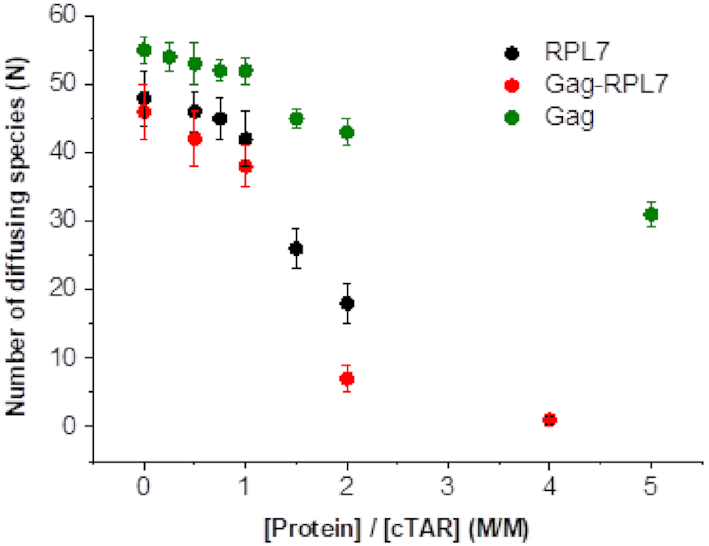
FCS analysis of the interaction between 100 nM TMR-5′-cTAR and increasing concentrations of Gag, RPL7 or Gag–RPL7 complex. Buffer was 50 mM Tris–HCl (pH 7.4), 150 mM NaCl, 1 mM MgCl_2_ and 1 mM DTT at 20°C.

Using a 100 nM TMR-5′-cTAR concentration, we found that in the absence of proteins, the number N of fluorescent cTAR molecules in the excitation volume was fully consistent with the theoretical number of molecules calculated from their concentration. Upon addition of increasing concentrations of either Gag, RPL7 or Gag–RPL7, we found only moderate changes in the number N of diffusing fluorescent species up to a protein/cTAR ratio of 1:1 (Figure 2, green, black and red points, respectively), as measured from the intercept of the autocorrelation curves in Supplementary Figure S2a, d and g. This absence of change of N indicated that no significant aggregation occurred under these conditions. In contrast, higher ratios (1.5:1, 2:1 and 5:1) induced a sharp drop in N indicating the formation of aggregates (Figure 2 and Supplementary Figure S2). As a consequence, we used a protein/DNA molar ratio of 1:1 in the further experiments.

Having defined the aggregation conditions, we next determined the binding parameters of Gag, RPL7 and Gag–RPL7 by titrating Fl-5′-cTAR by increasing concentrations of the proteins (Supplementary Figure S3). The titrations could be adequately fitted by Equation (2), assuming a 1:1 stoichiometry. Dissociation constants of 4 (±1) nM, 9 (±3) nM and 4.3 (±0.7) nM were obtained for Gag, RPL7 and Gag–RPL7, respectively. The *K*_d_ values of Gag for cTAR were similar to those previously described for other NA targets of Gag (45,60).

### Promotion of the cTAR/dTAR annealing kinetics by Gag, RPL7 and Gag–RPL7

**Figure 3.**
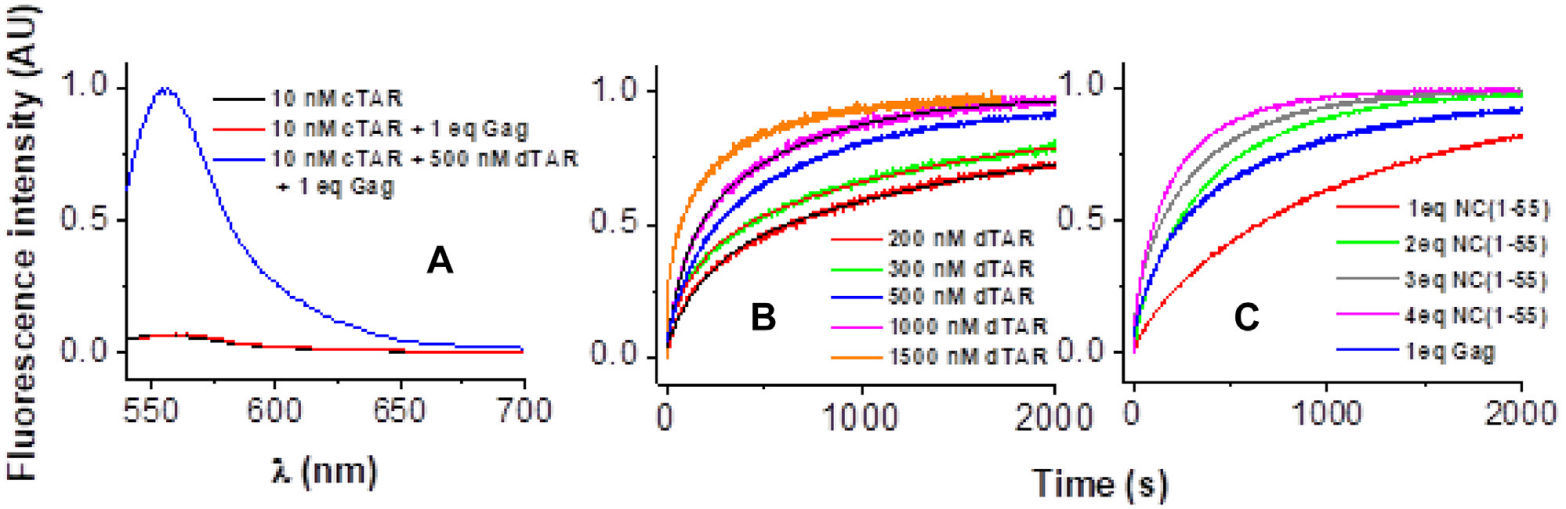
Fluorescence monitoring of the Rh6G-5′-cTAR-3′-Dabcyl-dTAR annealing reaction. (**A**) Emission spectra with excitation at 520 nm of 10 nM Rh6G-5′-cTAR-3′-Dabcyl (black), in the presence of Gag added at a protein/ODN molar ratio of 1 (red) and after completion of the reaction with 500 nM dTAR in the presence of Gag added at a protein/ODN molar ratio of 1 (blue). (**B**) Progress curves for the reaction of 10 nM doubly labeled cTAR with 200 (red), 300 (green), 500 (blue), 1000 (magenta) and 1500 nM (orange) dTAR in the presence of Gag added at a protein/ODN molar ratio of 1. The kinetic traces were fitted to Equation (2) to determine the *k*_obs1_ and *k*_obs2_ values. (**C**) Progress curves for the reaction of 10 nM doubly labeled cTAR with 500 nM dTAR in the presence of Gag added at a protein/ODN molar ratio of 1 (blue) or NC(1–55) added at a peptide: DNA molar ratio of 1:1, (red), 2: 1 (green), 3:1 (gray) or 4:1 (violet). Gag promotes hybridization with almost the same efficiency (*k*_obs1_ and *k*_obs2_ = 1.0 × 10^−2^ and 1.6 × 10^−3^ s^−1^, respectively) than that of NC(1–55) at a protein: ODN molar ratio of 2:1 (*k*_obs1_ and *k*_obs2_ = 1.1 × 10^−2^ and 2.1 × 10^−3^ s^−1^, respectively). All reported progress curves were averaged over two to four individual traces recorded using an excitation and emission wavelength of 520 and 555 nm, respectively. Buffer was 50 mM Tris–HCl (pH 7.4), 150 mM NaCl, 1 mM MgCl_2_ and 1 mM DTT at 20°C.

To characterize the cTAR/dTAR annealing mechanism promoted by Gag, RPL7 or their complex, we reacted under pseudo-first order conditions 10 nM Rh6G-5′-cTAR-3′-Dabcyl with, at least, a 10-fold molar excess of non-labelled dTAR in the presence of protein added at a molar ratio of 1:1. Due to the high affinity of Gag, RPL7 and Gag–RPL7 for cTAR, more than 90% of the proteins and oligonucleotides should be in their bound form in their mixture with cTAR and dTAR. At *t* = 0, the Rh6G fluorescence was strongly quenched by Dabcyl (Figure 3A, black spectrum) due to the proximity of the cTAR ends (61). During formation of the 55 bps ED, the distance between the probe and its quencher increased inducing a substantial recovery of Rh6G fluorescence (Figure 3A, blue spectrum).

A set of typical progress curves for the reaction of 10 nM doubly labeled cTAR with increasing dTAR concentrations in the presence of Gag is shown in Figure 3B. Completion of the reaction was reached in about 30 min for [dTAR] = 1000 nM, whereas more than 24 h was required in the absence of protein (49). The kinetic parameters governing the reaction were derived by fitting the fluorescence traces to the following double-exponential equation which satisfactorily describes the time course of the fluorescence increase (Figure 3B):

(3) $$\begin{eqnarray*} {I_t} &=& {I_f} - \left( {{I_f} - {I_0}} \right) \nonumber \\ &&\times\,\left( {ae_{{\rm}}^{( - k_{obs1} \times (t - {t_0}))} - \left( {1 - a} \right)\;e_{{\rm}}^{( - k_{obs2} \times {\rm{ }}(t - {t_0}))}} \right)\end{eqnarray*}$$

where *t_0_* is the dead time, *k*_obs1,2_ are the pseudo-first order rate constants of the reaction, *a*, the relative amplitude of the fast component and *I_t_*, *I_0_* and *I_f_* stand for the intensities of the actual, initial and final fluorescence intensity, respectively.

Since the HIV-1 nucleocapsid protein, NC(1–55), is a well-known NAC (49–50,62) that efficiently promotes cTAR/dTAR annealing, we compared the annealing potency of Gag to that of NC(1–55) (Figure 3C). Comparison of the annealing kinetics revealed that at a protein: ODN molar ratio of 1: 1, Gag promotes hybridization with an efficiency similar to that of NC(1–55) at a protein: ODN molar ratio of 2: 1.

**Figure 4.**
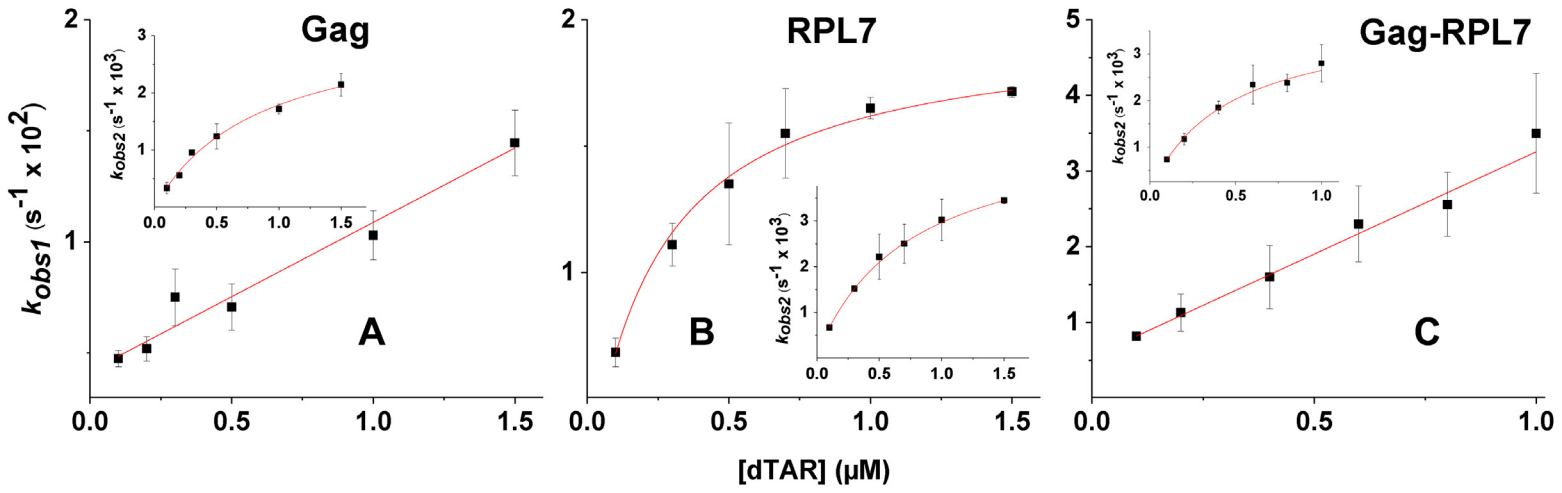
Dependence on dTAR concentration of the pseudo-first order rate constants, *k*_obs1_and *k*_obs2_, governing cTAR/dTAR annealing. The ODNs were reacted in the presence of Gag, RPL7 and the Gag–RPL7 complex using a protein/ODN molar ratio of 1:1. The *k*_obs1_and *k*_obs2_values were recovered from the fits of the kinetic traces, as described in Figure 3B, and then fitted to Equation (4) (straight red lines) or Equation (5) (red hyperboles). Each data point represents the mean ± standard error of the mean for at least three measurements. Buffer was as in Figure 3.

**Scheme 1.**
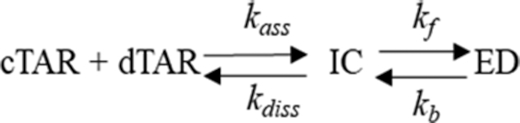
Two-step annealing mechanism.

In the presence of Gag, *k*_obs1_ and *k*_obs2_ values were linearly and curvilinearly dependent on [dTAR], respectively (Figure 4A), indicating that the annealing reaction proceeds via a two-step mechanism where the formation of the final stable ED is rate-limited by the slow conversion of a rapidly formed intermediate complex (IC) (Scheme 1):

IC formation and dissociation are governed by the second and first order rate constants *k*_ass_ and *k*_diss_, respectively. *k*_f_ and *k*_b_ are the first order rate constants for the IC conversion into ED and its reverse reaction, respectively. Scheme 1 predicts that:

(4) $$\begin{equation*}{k_{{\rm obs1}}} = {k_{{\rm ass1}}}\left[ {{\rm{dTAR}}} \right]{\rm{ }} + {k_{{\rm diss}}}\end{equation*}$$

and

(5) $$\begin{equation*}{k_{{\rm obs2}}} = \frac{{kfK_{a}^{*}\left[ {dTAR} \right]}}{{1 + K_{a}^{*}\left[ {dTAR} \right]}}\; + {k_{\rm b}}\end{equation*}$$

where *K*_a_* represents the equilibrium association constant that governs the IC stability. If *k*_ass_[dTAR] + *k*_diss_ > *k*_f_, then IC accumulates, leading to the hyperbolic variation of *k*_obs2_ observed in the inset of Figure 4A. Values of (6.6 ± 0.5) × 10^3^ M^–1^s^−1^ and (4.1 ± 0.2) × 10^−3^ s^–1^ were determined for the *k*_ass_ and *k*_diss_ rate constants, respectively (Table 1). Moreover, a (1.2 ± 0.2) × 10^6^ M^–1^ value was obtained for *K*_a*_, in agreement with the 1.6 × 10^6^ M^–1^ value calculated from the *k*_ass_/*k*_diss_ ratio. Finally, a value of (3.3 ± 0.6) × 10^−3^ s^–1^ was obtained for the sum of the forward (*k*_f_) and backward (*k*_b_) interconversion rate constants. The value of *k*_b_, given by the intercept, was very low (<0.0001 s^–1^), in line with the expected high stability of the ED, so that the (3.3 ± 0.6) × 10^−3^ s^–1^ value mainly corresponds to *k*_f_ (Table 1).

**Table 1.** Kinetic parameters for the Gag-promoted hybridization of cTAR to dTAR and its mutants

Compl Oligo	*k_ass_* (M^−1^s^−1^ × 10 ^-3^)	*k_diss_* (s^−1^ × 10^3^)	K_a_* (M^−1^ × 10^−6^)	*k*_*2*_(s^−1^ × 10^3^)	*k*_f_(s^−1^ × 10^3^)
°dTAR	6.6 ± 0.5	4.1 ± 0.2	1.2 ± 0.2		3.3 ± 0.6
**^¶^**dTAR	6.40 ± 0.04	1.6 ± 0.1	4.0 ± 0.3 ^#^		1.4 ± 0.1
**^¶^**dTAR-TL	5.4 ± 0.2	1.40 ± 0.05	3.8 ± 0.3^#^		0.80 ± 0.05
**^¶^**dTAR(1,2)	1.6 ± 0.2	0.090 ± 0.004	17.7± 0.6 ^#^		0.31 ± 0.06
**^¶^**dTAR-switch	4.9 ± 0.05	0.62 ± 0.02	7.9 ± 0.04^#^	0.52 ± 0.01	0.10 ± 0.02

°Constants obtained by the analytical approach.

^¶^Constants obtained with Dynafit (numerical approach).

^#^Calculated from *k*_ass_/*k*_diss_.

**Scheme 2.**
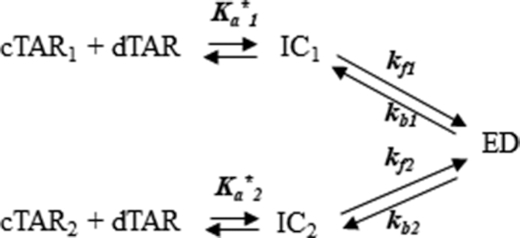
Annealing mechanism with two parallel pathways.

Next, the RPL7- and Gag–RPL7-promoted cTAR/dTAR annealing reactions were investigated using the same protocol. As for Gag, all recorded progress curves were adequately described by Equation (2). In the presence of RPL7 alone, both *k*_obs1_ and *k*_obs2_ values hyperbolically vary with [dTAR] (Figure 4B), indicating that RPL7 promotes ED formation through two parallel and rate-limited pathways as illustrated by Scheme 2:

where, *K*_a_*_1_ and *K*_a_*_2_ represent the equilibrium association constants governing the stability of the intermediate species IC_1_ and IC_2_, respectively, that both convert into the unique final ED. A similar mechanism with two parallel pathways was already reported for the cTAR/dTAR hybridization promoted by peptide E from the core protein of hepatitis virus C (51). The kinetic parameters are reported in Table 2. As for Gag, a possible ED dissociation could not be evidenced from our data (*k*_b_ < 5 × 10^−5^ s^−1^), suggesting that RPL7 is unable to dissociate ED.

**Table 2.** Kinetic parameters for the RPL7-promoted hybridization of cTAR to dTAR and its mutants

Compl Oligo	*k_ass1_* (M^−1^s^−1^ × 10 ^-3^)	*k_ass2_* (M^−1^s^−1^ × 10 ^-3^)	*k_diss1_* (s^−1^ × 10^3^)	*k_diss2_* (s^−1^ × 10^3^)	K_a_*_1_ (M^−1^ × 10^−6^)	K_a_*_2_ (M^−1^ × 10^−6^)	*k_f1_* (s^−1^ × 10^3^)	*k_f2_* (s^−1^ × 10^3^)
°dTAR	nd	nd	nd	nd	5.2 ± 0.3	1.5 ± 0.1	19.3 ± 0.5	5.1 ± 0.2
**^¶^**dTAR	9.9 ± 0.2	2.0 ± 0.1	2.7 ± 0.1	3.4 ± 0.5	3.7 ± 0.2^#^	0.6 ± 0.1^#^	19.3 ± 0.5	3.3 ± 0.1
**^¶^**dTAR-TL	7.8 ± 0.2	1.0 ± 0.07	3.2 ± 0.1	2.1 ± 0.7	2.4 ± 0.1^#^	0.5 ± 0.2^#^	13 ± 2	2.9 ± 0.2
**^¶^**dTAR(1,2)	6.20 ± 0.03		0.8 ± 0.1		7.6 ± 1^#^		0.50 ± 0.01	
**^¶^**dTAR-switch	6.9 ± 0.9	3.0 ± 0.1	20 ± 5	0.20 ± 0.04	0.34 ± 0.1^#^	15 ± 3^#^	12 ± 1	0.85 ± 0.03

°Constants obtained by the analytical approach.

nd, not determinable by this approach.

^¶^Constants obtained with Dynafit (numerical approach).

^#^Calculated from *k*_ass_/*k*_diss_.

As for Gag alone, we observed with the Gag–RPL7 complex a linear and hyperbolic variation of *k*_obs1_ and *k*_obs2_, respectively, indicating that the complex also promotes cTAR/dTAR annealing according to Scheme 1 (Figure 4C). The kinetic data are reported in Table 3. A value of (5.3 ± 0.1) × 10^6^ M^−1^ was found for *K_a_*, in good agreement with the *k*_ass_/*k*_diss_ ratio (5.2 × 10^6^ M^−1^).

**Table 3.** Kinetic parameters for the Gag–RPL7-promoted hybridization of cTAR to dTAR and its mutants

Compl Oligo	*k*_ass_ (M^−1^s^−1^ × 10 ^-3^)	*k*_diss_ (s^−1^ × 10^3^)	*K*_a_* (M^−1^ × 10^−6^)	*k*_f_ (s^−1^ × 10^3^)
°dTAR	28 ± 1	5.3 ± 0.1	5.3 ± 0.1	3.8 ± 0.2
**^¶^**dTAR	25.4 ± 0.2	5.0 ± 0.4	5.1 ± 0.4^#^	3.2 ± 0.2
**^¶^**dTAR-TL	17.5 ± 0.9	4.0 ± 0.3	4.4 ± 0.5^#^	1.20 ± 0.05
**^¶^**dTAR(1,2)	11.1 ± 0.1	3.7 ± 0.2	3.0 ± 0.2^#^	2.1 ± 0.3
**^¶^**dTAR-switch	4.6 ± 1	0.57 ± 0.05	8.0 ± 2^#^	0.3 ± 0.05

°Constants obtained by the analytical approach.

^¶^Constants obtained with Dynafit (numerical approach).

^#^Calculated from *k*_ass_/*k*_diss_.

To further validate both the kinetic models and rate constants, the experimental traces were analysed with the numerical solving software Dynafit (63). A typical analysis of the Gag-assisted cTAR/dTAR kinetic traces is represented in Supplementary Figure S4. Examination of Tables 1–3 indicates that the values calculated by Dynafit from the set of progress curves satisfactorily agree with those obtained by the analytical approach confirming that Schemes 1 and 2 adequately describe the reaction mechanisms. The advantage of the numerical approach over the analytical one is that it makes no *a priori* on the respective values of the kinetic rate constants. Moreover, this approach allows also to recover the association and dissociation rate constants of the two pathways observed with RPL7, giving a more complete picture of the reaction scheme. For these reasons, we used the kinetic rate constants recovered from the numerical approach to compare the effect of the proteins.

Comparison of the parameters governing the fastest pathway promoted by RPL7 (Table 2) to those of the unique pathway of Gag (Table 1) revealed that the two proteins promote the IC formation at the same velocity, but that RPL7 converts the IC to the ED ∼ 13-fold faster than Gag, confirming that RPL7 stimulates cTAR/dTAR hybridization more efficiently than Gag (48). Further comparison with Gag–RPL7 (Table 3) indicates that the complex stimulates the IC formation three to four times faster than the isolated proteins. In contrast, the interconversion rate is only 2-fold higher than that of Gag alone. As the mechanism of Gag–RPL7 is similar to that of Gag and as the IC conversion rate of Gag–RPL7 is close to that of Gag, this strongly suggests that the mechanism of the complex is dominated by Gag.

### Oligonucleotide destabilizing activity of Gag, RPL7 and their complex

**Figure 5.**
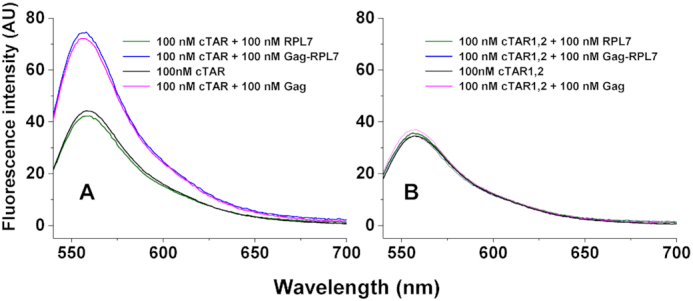
Destabilizing activity of Gag, RPL7 and Gag–RPL7 on cTAR (**A**) and cTAR1,2 (**B**). The destabilizing activity was evaluated by comparing the fluorescence spectra of 100 nM doubly labelled oligonucleotide in the absence (black) and in the presence of an equimolar concentration of Gag (magenta), RPL7 (green) or their complex (blue). Buffer was 50 mM Tris–HCl (pH 7.4), 150 mM NaCl, 1 mM MgCl_2_ and 1 mM DTT at 20°C.

To further characterize the mechanism by which Gag, RPL7 and Gag–RPL7 stimulate cTAR/dTAR annealing, we determined their ability to destabilize the stems of the bound oligonucleotides. To this aim, we examined the effect of the proteins added at equimolar concentration on the emission spectrum of the doubly labeled cTAR or cTAR1,2, an ∼4-fold more stable mutant lacking the terminal bulges (64) (Figure 1). To favor the formation of the protein/ODN complexes, a 100-nM oligonucleotide concentration (10-fold higher than that used in the kinetic measurements) was used. Figure 5A shows a substantial enhancement of the doubly labelled cTAR emission upon addition of Gag, in agreement with an increase of the inter-chromophore distance likely due to the destabilization of the stem end. In contrast, no fluorescence increase was observed for RPL7 alone (Figure 5A), suggesting that the annealing activity of RPL7 does not involve NA destabilizing properties. Similar spectra were recorded for the cTAR/Gag and cTAR/Gag–RPL7 mixtures (Figure 5A), indicating that Gag–RPL7 and Gag alone present the same NA destabilizing potency, confirming the predominant role of Gag in the complex. In a next step, we repeated the same measurements with cTAR1,2. The spectrum of the free oligonucleotide was superimposable to those of its mixtures with Gag, RPL7 and Gag–RPL7 (Figure 5B), indicating that none of the proteins is able to melt the stable stem end of cTAR1,2.

### Mutational study of the cTAR/dTAR annealing promoted by Gag, RPL7 and Gag–RPL7

Next, to further decipher the annealing mechanisms, we investigated the impact of the sequence and stability of dTAR on the Gag-, RPL7- and Gag–RPL7-promoted cTAR/dTAR annealing kinetics. To this aim, we studied, as above, the reaction of the doubly labeled cTAR with a series of three dTAR mutants (Figure 1) in the presence of Gag, RPL7 or Gag–RPL7. The sets of progress curves were analysed by the Dynafit software (Tables 1–3).

**Figure 6.**
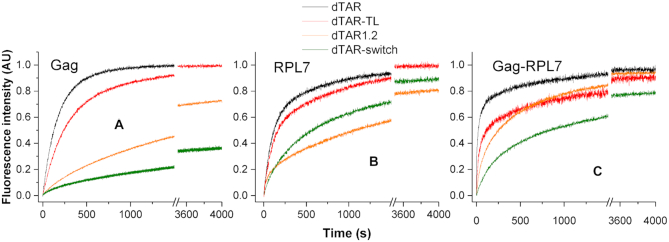
Effect of dTAR sequence and stability on the kinetics of its annealing with cTAR promoted by Gag (**A**), RPL7 (**B**) and Gag–RPL7 (**C**). Kinetic traces for the reaction of 10 nM doubly labeled cTAR with 300 nM non-labeled dTAR (black curves), dTAR-TL (red curves), dTAR-switch (green curves) or dTAR1.2 (orange curves) in the presence of Gag, RPL7 or Gag–RPL7. Experimental conditions were as in Figure 3. Excitation and emission wavelengths were at 520 and 555 nm, respectively, to monitor the Rh6G emission. The traces were fitted to Equation (2), except for the trace of the Gag-promoted cTAR/dTAR-switch reaction that was fitted to a triple-exponential function. The values of the kinetic parameters governing the different reactions are listed in Tables 1–3. A protein/ODN molar ratio of 1:1 was used in all cases.

First, dTAR was substituted by the dTAR-TL mutant where the six nucleotides of the loop were replaced by T residues to prevent base-pairing with the cTAR loop (Figure 1). As with the wild-type cTAR/dTAR system, we found that the cTAR/dTAR-TL reaction is adequately described by Scheme 1 in the presence of Gag and Gag–RPL7 and by Scheme 2 in the presence of RPL7 alone. Figure 6A shows a slightly reduced velocity of the Gag-promoted reaction with dTAR-TL. From Table 1, we observe that the loop mutation has almost no impact on the *k*_ass_ and *k*_diss_ values, but reduces by ∼2-fold the *k*_f_ value. This suggests that the loop is marginally involved in the Gag-directed IC formation, but contributes to its conversion into the ED. For RPL7, both the progress curves (compare black curve for cTAR/dTAR and red curve for cTAR/dTAR-TL in Figure 6B) and the kinetic parameters (Table 2) indicate that the contribution of the loops is moderate in the two pathways promoted by RPL7. The mutation of the loop induces only a slight decrease (35%) in the stability of the IC and the *k*_f1_ value in the fast pathway. For the slow pathway, the changes are even less. Finally, with the Gag–RPL7 complex (Figure 6C and Table 3) as for Gag, the main effect of the loop mutation is a 2.5-fold decrease in the *k_f_* value, indicating that the loop is involved in the IC conversion to ED and confirming that the mechanism of Gag–RPL7 is dominated by Gag.

The contribution of the stems was examined by replacing dTAR by the dTAR1.2 mutant, which is 6-fold more stable (*ΔG* = −12.6 kcal.mol^−1^) than dTAR (*ΔG* = −2.1 kcal.mol^−1^) as calculated with mFold, due to the introduction of two bases (at positions 51 and 54) conferring a perfect double stranded structure to the bottom part of dTAR stem (Figure 1). In the presence of Gag or RPL7, the reaction of cTAR with dTAR1,2 was considerably slowed down as compared to that with dTAR (Figure 6A and B, compare orange and black curves), whereas a more limited effect was observed for the Gag–RPL7-promoted reaction (Figure 6C). Analysis of the kinetic parameters shows that in the presence of Gag, the replacement of dTAR by dTAR1,2 does not change the reaction scheme but drastically modifies the values of the kinetic rate constants (Table 1). This replacement decreases the *k*_ass_ value by a factor of 4, but at the same time also decreases the *k*_diss_ value by 18-fold, inducing a 4-fold increase in the IC stability. Thus, the increased stability of dTAR1,2 slows down the Gag-promoted formation of the IC and stabilizes the IC. This strongly suggests that similarly to NC (49,50), the Gag-promoted IC is nucleated through the stem ends. The increased stability of dTAR1,2 also slows down the IC conversion into ED, as shown by the 5-fold decrease in the *k_f_* value. In the case of RPL7, the reaction scheme with two parallel pathways was converted into a simple two-step mechanism when dTAR was replaced by dTAR1,2. This suggests that one of the two pathways became negligible as a result of the increased stability of dTAR1,2. Comparison of the kinetic parameters of this two-step pathway with those of the fast pathway with dTAR reveals that the cTAR/dTAR1,2 IC is stabilized by 2-fold as a result of a 3-fold decrease in the *k*_diss_ value associated to a 1.6-fold decrease in the *k*_ass_ value (Table 2). Moreover, the high stability of dTAR1,2 has a major impact on the RPL7-promoted IC conversion, as can be seen from the nearly 40-fold decrease of the *k*_f_ value (Table 2). Thus, similarly to Gag, the RPL7-promoted annealing of cTAR with dTAR is probably nucleated through the stems and strongly depends on their stability. In the presence of the Gag–RPL7 complex, the replacement of dTAR by dTAR1,2 decreases by 2.5-fold the *k*_ass_ value, suggesting that as for the two proteins alone the annealing is initiated through the stem ends. This decrease in the *k*_ass_ value was accompanied by a slight decrease (1.35-fold) of the *k*_diss_ value, resulting in only a moderate change in the IC stability (Table 3). Comparison of the RPL7/Gag complex with Gag in their ability to promote the cTAR/dTAR1,2 reaction revealed that the complex increased the *k*_ass_ and *k*_f_ values by 7-fold and the *k*_diss_ value by 40-fold. Thus, the two proteins clearly exert a synergistic role by promoting the formation and dissociation of the IC as well as the IC conversion to ED, suggesting a key role of RPL7 to help Gag in annealing stable NA sequences during viral replication.

**Scheme 3.**
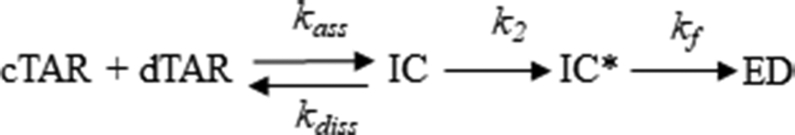
Three-step annealing mechanism.

The involvement of the stems in the annealing reaction was further investigated by replacing dTAR by dTAR-switch, a mutant where the three terminal base pairs are inverted. This replacement induced a substantial decrease of the speed of the Gag-promoted annealing reaction (Figure 6A, green curve). Surprisingly, the progress curves were best described by a triple-exponential function, which suggests that the cTAR/dTAR-switch annealing reaction likely requires an additional step, as illustrated by Scheme 3:

This three-step mechanism involves the fast accumulation of a pre-equilibrium intermediate complex IC, slowly rearranging into a transient species IC*, which then converts into ED. The kinetic traces (Supplementary Figure S5) were well fitted using Scheme 3 and the kinetic parameters in Table 1. Interestingly, the replacement of dTAR by dTAR-switch does only marginally impact the formation of the IC, but dramatically slows down its conversion into ED, through two rate-limiting steps (*k*_2_ = 0.52 × 10^−3^ s^−1^ and *k*_f_ = 0.1 × 10^−3^ s^−1^) (Table 1).

With RPL7, the replacement of dTAR by dTAR-switch was found to preserve the mechanism with two parallel pathways. For the fast pathway, we observed a limited 30% decrease of the *k*_ass1_ value accompanied by a strong destabilization of the IC (by one order of magnitude) due to a drastic increase in the *k*_diss1_ value. Moreover, the rate constant of the IC conversion into ED was decreased by about 40% as compared to that observed with dTAR. In contrast, for the slow pathway, we observed a stabilization by one order of magnitude of the IC accompanied by a four-fold decrease in its conversion rate into the ED (Table 2).

With the Gag–RPL7 complex, the kinetic rate constants and stability of the cTAR/dTAR-switch IC are fully matching with those obtained with Gag, indicating that IC formation is governed by Gag. In contrast, the presence of RPL7 in the protein complex allows a one-step conversion of the IC into the ED, albeit with a rather slow rate constant (*k*f = 0.3 × 10^−3^ s^−1^). Thus, RPL7 cooperates with Gag to facilitate the conversion step in this case. Further comparison with dTAR data revealed that the significantly slower annealing process observed with dTAR-switch (Figure 6C) is related to an ∼6- and ∼10-fold decrease in the rate of IC formation and conversion, respectively (Table 3). As for cTAR1,2, this confirms the key contribution of the stem ends for the Gag–RPL7-assisted reaction.

### Arrhenius analysis of the cTAR/dTAR annealing reaction

**Figure 7.**
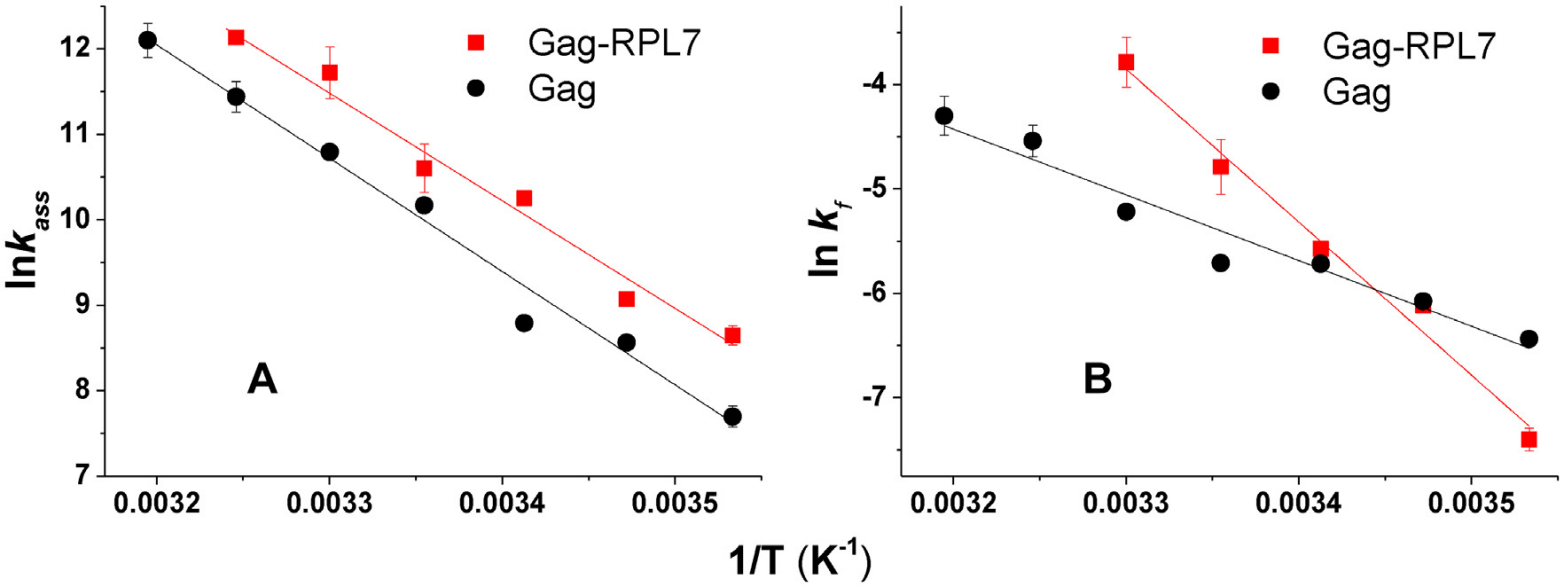
Arrhenius plots for the cTAR/dTAR annealing kinetics in the presence of Gag (black), and Gag–RPL7 (red) at a protein/ODN molar ratio of 1:1. Temperature dependence of *k*_ass_ (**A**) and *k*_f_ (**B**) for the reaction of 10 nM doubly labeled cTAR with 100 nM dTAR. The solid lines were generated with Equation (6) and the best estimates of the activation energies *E*_a1_ and *E*_a2_, respectively given in Table 4. It was checked that no aggregation occurred on changing the temperature.

To further investigate the two-step mechanism of Gag- and Gag–RPL7-promoted cTAR/dTAR annealing reaction, we examined the temperature dependence of the kinetic rate constants through an Arrhenius plot (Figure 7). The same approach could not be applied to the two parallel two-step pathways of the RPL7-promoted reaction, due to the too large number of rate constants in this case. According to the Arrhenius model, the thermodynamic parameters for the transition state can be derived from the reaction rates using:

(6) $$\begin{equation*}{\rm{ln}}\,{k_{1,2}} = - {E_{a1,2}}/R \times 1/T + {\rm{ln}}\,A\end{equation*}$$

where the rate constant *k*_1,2_ corresponds to *k*_ass_ (given by *k*_obs1_/[cTAR]) and *k*_f_, respectively, *A* is a pre-exponential factor, *E*_a1,2_ is the activation energy, *R* is the universal gas constant and *T* is the absolute temperature in Kelvin.

The values of the activation energies *E*_a1,2_ and the corresponding transition state enthalpies Δ*H*_1,2_ are listed in Table 4. For the two systems, the *k*_ass_ and *k*_f_ values increased with the temperature. Above 30°C, the kinetic traces recorded with Gag–RPL7 became mono-exponential. The slopes of the straight lines drawn through the data of Figure 7A (*k*_ass_) for Gag alone and its complex with RPL7 are close (26 kcal.mol^−1^.K^−1^), indicating that the first step of the annealing reactions is characterized by similar energy barriers with the two protein systems, confirming the dominant role of Gag in the first step of the Gag–RPL7-directed annealing process. Analysis of the temperature dependence of *k*_f_ (Figure 7B) indicates that the second step of the Gag–RPL7-stimulated reaction is characterized by a higher energy barrier (28 kcal.mol^−1^.K^−1^) than that of the reaction promoted by Gag alone (12.3 kcal.mol^−1^.K^−1^). From the calculated Δ*H*_1,2_ values (Table 4), we deduced (65–67) that for Gag alone and the Gag–RPL7 complex, a melting of ∼ 5–6 bps is required for IC formation while ∼ 3 and 5–6 bps, respectively need to be melted for the IC conversion into ED.

**Table 4.** Energy of activation (*E*_a1,2_) and transition state enthalpy (*ΔH*_1,2_) for the cTAR/dTAR annealing reactions promoted by Gag and the Gag–RPL7 complex

Protein	*E*_a1,2_* (kcal.mol^−1^)	*ΔH*_1,2_** (kcal.mol^−1^)
Gag	26.5 ± 2	26
	12.3 ± 1	11.7
Gag–RPL7	25 ± 2	24.4
	28 ± 2	27.4

*Determined from the data of Figure 7.

**Calculated from *ΔH = Ea-RT* with *T* = 293 K.

### RPL7 boosts the Gag-promoted tRNA^Lys^_3_/(+)PBS annealing

As RPL7 was shown above to help Gag in promoting the annealing of stable sequences, we next investigated the possible role of RPL7 in the Gag-promoted annealing of the physiologically relevant tRNA^Lys^_3_/(+)PBS system. The annealing of the stable tRNA^Lys^_3_ to (+)PBS RNA in the absence of protein has been previously shown to be extremely slow both at room temperature and 37°C (47,68–69).

**Figure 8.**
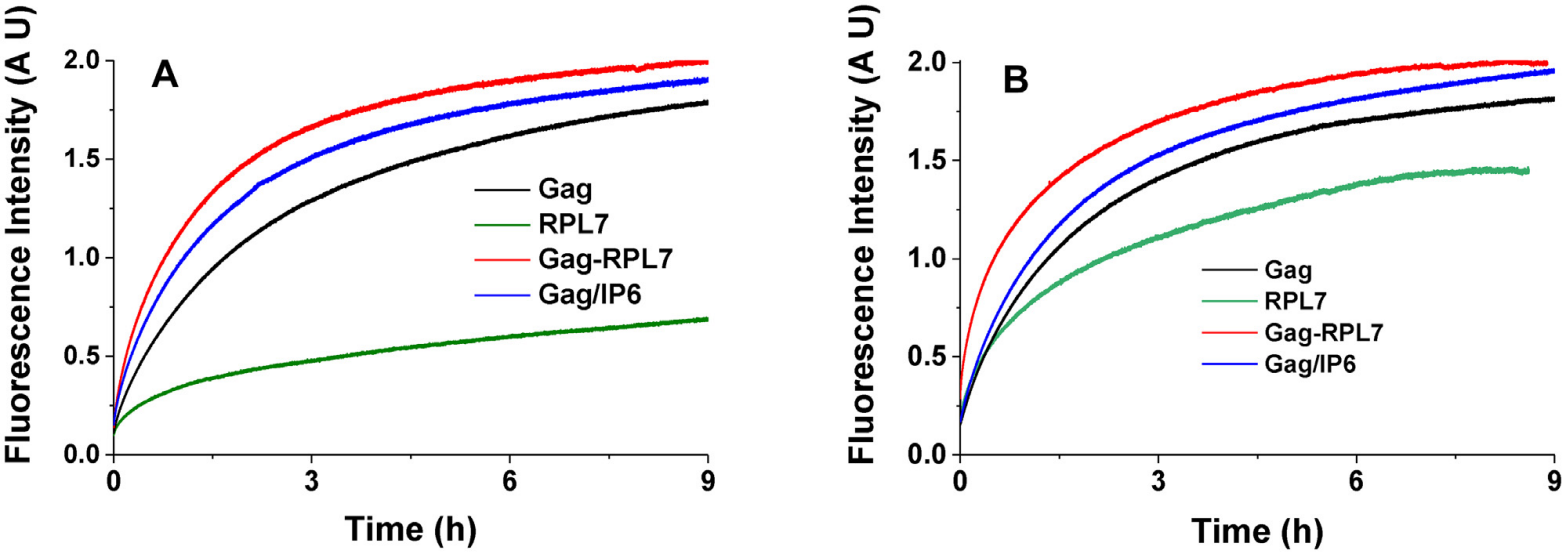
Progress curves for the reaction of 10 nM Rh6G-5′-(+)PBS RNA-3′-Dabcyl with 700 nM tRNA^Lys^_3_ in the presence of Gag (black and blue), RPL7 (green) and Gag–RPL7 (red) added at a protein/ODN ratio of 1:1 (**A**) and 2:1 (**B**). The kinetics with Gag were done either in the absence (black) or in the presence of 20 μM IP6 (blue). Buffer: 50 mM Tris–HCl (pH 7.4), 150 mM NaCl, 1 mM MgCl_2_ and 1mM DTT at 20°C. The fluorescence emission of Rh6G was followed at 555 nm with excitation at 520 nm.

We first performed the annealing reaction in the same conditions as with the cTAR/dTAR system, by adding Gag, RPL7 and Gag–RPL7 at a 1/1 molar ratio to the oligonucleotides (Figure 8A). While RPL7 alone was highly inefficient in promoting the annealing reaction, the Gag–RPL7 complex was found to increase the observed kinetic rate constants by a factor of 3 as compared to Gag alone (Table 5). Since tRNA^Lys^_3_ (76 nts) is significantly longer than the TAR species (55 nts), we then determined whether the annealing reaction would be faster at a higher protein to oligonucleotide molar ratios (Figure 8B and Table 3). Interestingly, the kinetic constants with Gag did not change significantly when the Gag/ODN molar ratio was raised from 1:1 to 2:1. In contrast, the same change in the molar ratio induced an about two-fold increase in the observed rate constants for both RPL7 and Gag–RPL7. As a result, at a 2:1 molar ratio a 6- to 8-fold increase in the *k*_obs1_ and *k*_obs2_ values was observed with the Gag–RPL7 complex as compared to Gag (Table 5), clearly confirming that RPL7 can boost the Gag-promoted tRNA^Lys^_3_/(+)PBS annealing reaction. Finally, we compared RPL7 with IP6 that was previously shown to activate the Gag-promoted tRNA^Lys^_3_ /(+)PBS RNA annealing reaction (10). In our conditions, IP6 was observed to promote the Gag chaperone activity but somewhat less efficiently than RPL7. Moreover, in contrast to RPL7, IP6 does not induce a further increase in the annealing speed at 2:1 Gag/ODN molar ratio compared to 1:1 molar ratio (Table 5), suggesting that RPL7 and IP6 may have different mechanisms.

**Table 5.** Kinetic parameters for the hybridization of (+)PBS RNA to tRNA^Lys^_3_ promoted by Gag, RPL7, Gag–RPL7 and Gag/IP6

Proteins	Protein/ODN ratio	*k*_obs1_ (s^−1^ × 10^4^)	*k*_obs2_ (s^−1^ × 10^5^)
**Gag**	1	1.5 ± 0.1	2.2 ± 0.4
	2	1.6 ± 0.1	1.4 ± 0.4
**RPL7**	1	2.1 ± 0.9	0.7 ± 0.2
	2	3.3 ± 0.2	1.9 ± 0.6
**Gag–RPL7**	1	4 ± 1	7.5 ± 1
	2	9 ± 1	11 ± 1
Gag/IP6*	1	2.7 ± 0.5	4.1 ± 0.7
	2	2.4 ± 0.3	3.5 ± 0.2

*IP6 was added at 20 μM.

## DISCUSSION

In HIV-1 infected cells, RNA dimerization and virus assembly are orchestrated by the structural polyprotein precursor Gag. During these steps, Gag is thought to rearrange NAs into their most thermodynamically stable conformation due to its NAC activity (7,14,24,43,70). However, since Gag exhibits limited NAC activity, it is believed that interaction with cellular partners, such as IP6 and RPL7 can compensate for this weakness (10). Here, we investigated and compared the mechanisms by which Gag, RPL7 and Gag–RPL7 anneal the canonical stem-loop cTAR and dTAR complementary DNA sequences, often used to investigate the chaperone properties of viral proteins (49–52,71). In the high salt and low protein/oligonucleotide ratio conditions used in our assay, both proteins were found to bind to cTAR with nanomolar affinity to mainly one binding site.

The Gag-promoted reaction was found to be nucleated from the stem ends as a consequence of its NA destabilization properties (Figure 5). In line with the reported preferential binding of mature NC on cTAR (72) and TAR (73) to G residues close to mismatches in the lower part of the stem, our data suggest that Gag preferentially binds to the same binding site through its NC domain. The reaction then proceeds via a two-step kinetic pathway which includes the formation of a fast pre-equilibrium intermediate and its rate-limited conversion into the final ED. As for Tat (52) and NCp7 (49,50), the Gag-promoted annealing pathway differs from the one in the absence of protein which proceeds through a kissing complex intermediate (50,74), indicating that Gag modifies the annealing mechanism. The Arrhenius plot suggests a melting of about 5–6 bps for the fast kinetic component, suggesting that the reactive species likely corresponds to cTAR species where the terminal double stranded segment is melted. This melting has been shown to occur spontaneously at room temperature (29,58,64), but may be further promoted by Gag (Figure 5). This destabilizing activity of Gag is likely related to its NC domain, since the mature NCp7 induces a similar limited melting when added at low (2:1) ratio to cTAR (28). A more extended melting of the cTAR stem was observed when NCp7 was added at saturating concentrations (10:1 molar ratio) (28,64). Such a high molar ratio could unfortunately not be tested with Gag, as it starts precipitating cTAR molecules at molar ratios above 1:1 (Figure 2). The IC formation likely relies also on the Gag-induced attraction between the Gag/cTAR and Gag/dTAR complexes, which facilitates the diffusional search for the complementary sequences. Further conversion of the IC into the ED requires the melting of three additional base pairs, as suggested by the activation energy associated to the conversion rate constant (Table 4). Interestingly, Gag added to cTAR and dTAR at a 1:1 molar ratio was found to exhibit the same annealing activity than NCp7 added at a 2:1 molar ratio, using a similar reaction pathway mainly initiated through the stem ends and leading to an IC of similar stability (50). This suggests that the NC domain of Gag is not the only one that contributes to Gag's NAC activity at this molar ratio and that the MA sequence, which was reported to bind to NAs through its basic domain, may also contribute to the Gag-induced attraction between Gag–cTAR and Gag–dTAR complexes. In contrast, when Gag and NCp7 were used at higher molar ratios, the mature NCp7 protein was found to be a much more efficient chaperone than Gag (10), indicating that the MA domain limits Gag's chaperone activity at these ratios. This suggests that the role of Gag domains during viral assembly is finely regulated by the concentration of Gag linked to its NA targets and by co-factors such as IP6 which interact directly with these domains.

**Figure 9.**
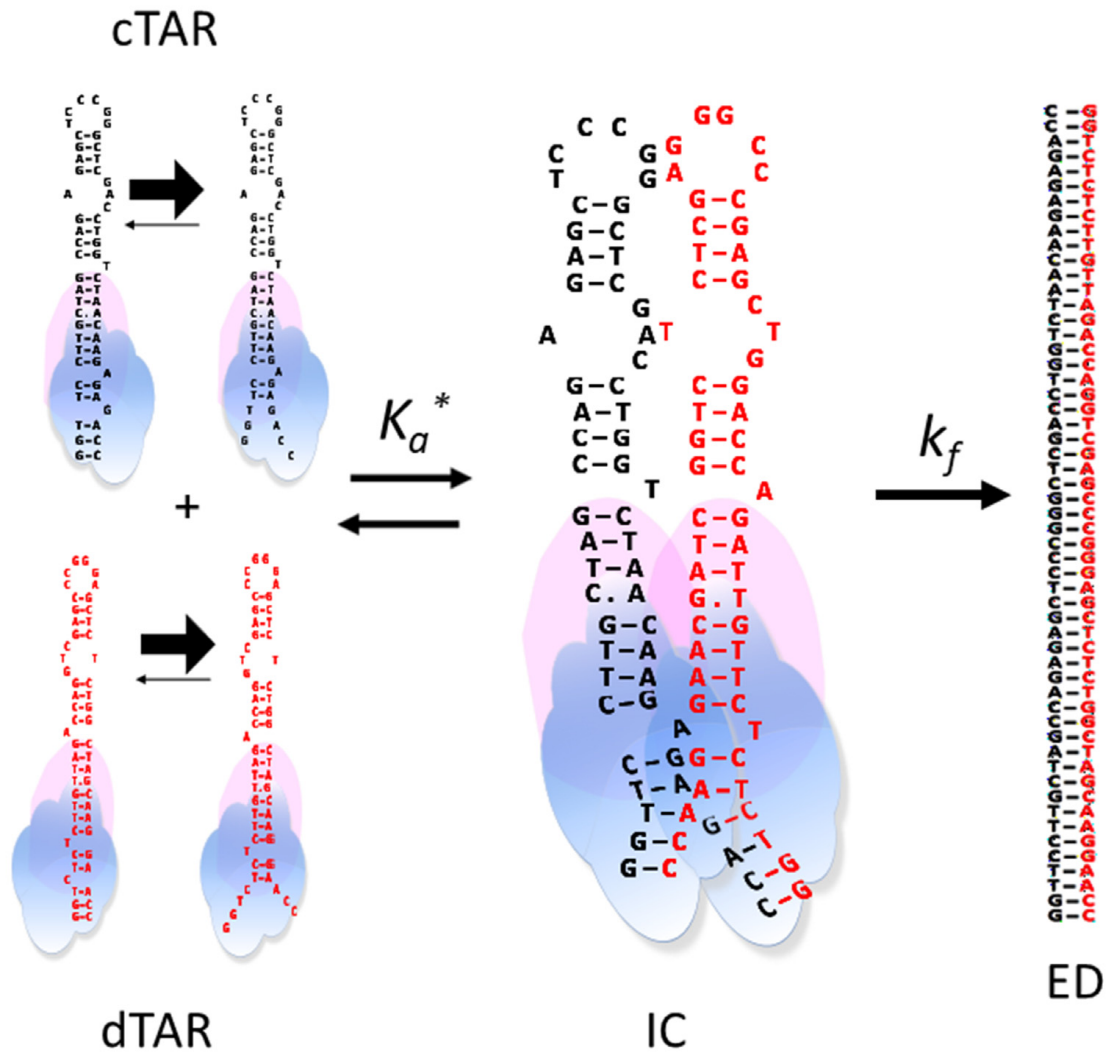
Proposed reaction scheme for the Gag- and Gag–RPL7-promoted cTAR/dTAR annealing at a 1:1 protein:oligonucleotide molar ratio. In the presence of Gag alone (represented by a blue cloud) or its complex with RPL7 (represented by a pink cloud), the reaction proceeds through a two-step pathway, with the formation of an intermediate complex IC which is further converted into the ED. Due to the destabilizing properties of Gag and Gag–RPL7, the equilibrium due to thermal fraying between the closed and partially opened oligonucleotides is displaced to the right, leading to the fast formation of the IC. Its slow conversion into the final ED requires an additional melting of 3–6 bps.

Based on our data, a mechanism for the Gag-promoted cTAR/dTAR annealing can be suggested (Figure 9). Nucleation of the IC likely requires the melting of the lower double-stranded segment of cTAR and dTAR induced both by temperature and the destabilization activity of Gag. The simultaneous destabilization of both cTAR and dTAR sequences may not be compulsory, since dTAR1,2 which is not destabilized by Gag (Figure 5) can also react with cTAR. The destabilization of cTAR and its close proximity with dTAR1,2 permitted by the NA aggregating properties of Gag are likely sufficient in this case to promote but with a low efficiency (as shown by the decrease in the *k*_ass_ value) the strand exchange that nucleates the IC (75). Then, further melting of 3 bps triggers the cTAR/dTAR IC conversion into the ED. This step is likely facilitated by the cTAR bulge at position 49 and the corresponding bulge at position 7 in dTAR, because the replacement of dTAR by dTAR1,2 where this bulge is absent leads to a strongly decreased conversion rate. In addition, the absence of this bulge probably hinders the base fluctuations needed for the dissociation of the IC, explaining the high stability of the cTAR/dTAR1,2 IC. Since the conversion step was slowed down with the dTAR-TL mutant, this step is likely also facilitated by the loops, which may help to properly position the reacting species, presumably by loop-loop contacts. For the Gag-promoted annealing of cTAR with dTAR-switch, the strong similarity of the *k*_ass_ value with that of cTAR/dTAR suggests that IC nucleation is similar in both systems, involving the nucleotides of the last double-strand segment of the stem. However, as this segment is inverted in dTAR-switch as compared to dTAR, the upper parts of the stem are not properly positioned in the IC to nucleate the ED, explaining the decreased *k*_f_ value observed with dTAR-switch as compared to dTAR. The additional rate-limiting step for the conversion of the cTAR/dTAR-switch IC into ED likely corresponds to IC rearrangements required to dissociate the base pairs between the stem ends and initiate base pairing in the upper parts of the reactants.

**Figure 10.**
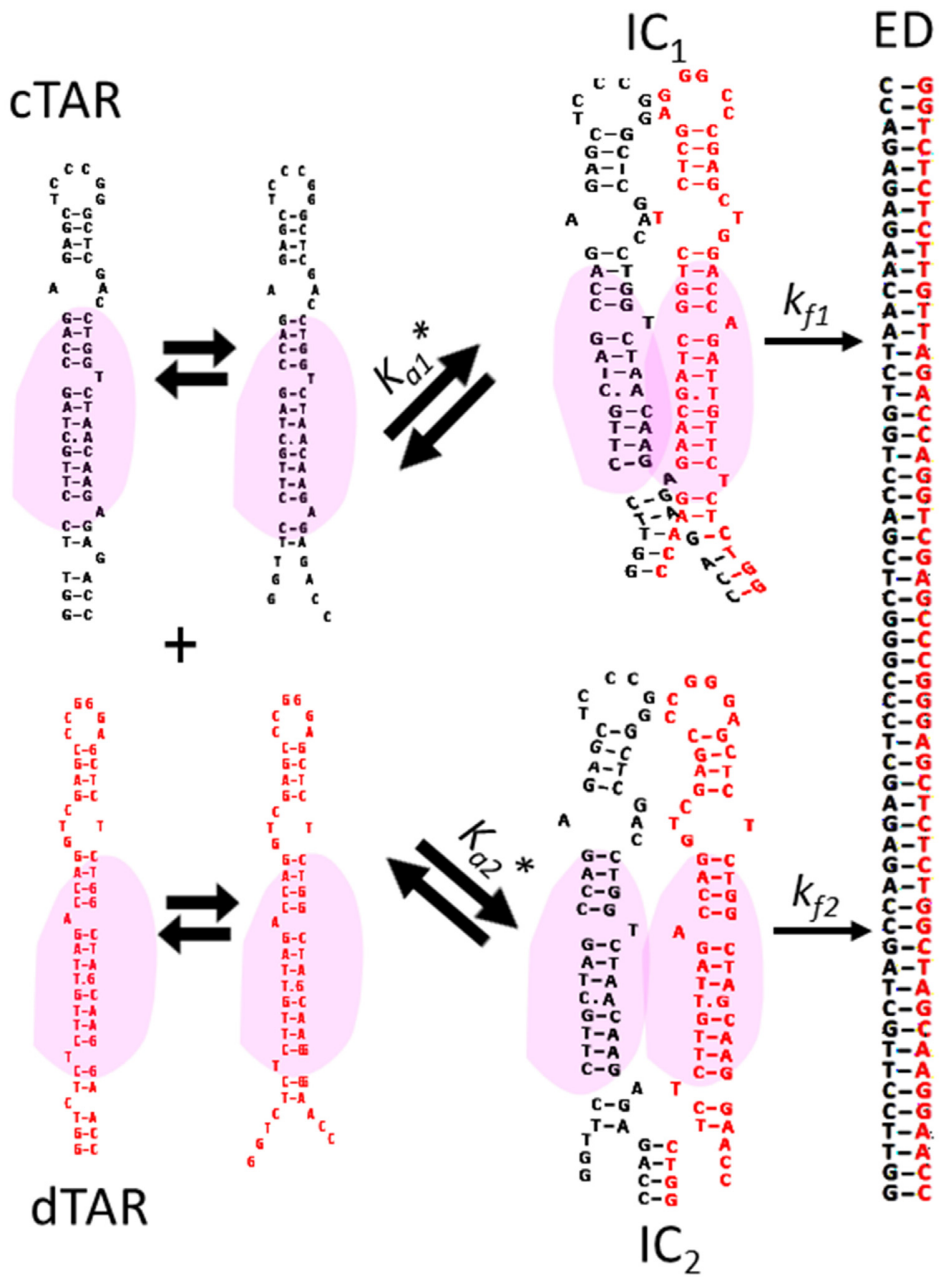
Proposed reaction scheme for the RPL7-promoted cTAR/dTAR annealing. In the presence of RPL7 (represented by a pink cloud) added at a 1:1 molar ratio, the annealing reaction is thought to proceed via two parallel two-step pathways. The fast pathway likely relies on a ‘zipper’ mechanism similar to that observed for Gag alone and Gag–RPL7 (Figure 9). The slow pathway likely relies on an ‘invasion’ mechanism where a RPL7-bound cTAR or dTAR species melted by temperature may invade a closed RPL7-bound complementary sequence. This invasion leads to the IC2 stabilized by only 4 base pairs, which converts more slowly than IC1 into the ED (Table 2).

For the RPL7-mediated cTAR/dTAR annealing, the annealing process was found to involve two parallel kinetic pathways which are nucleated from the ODN stem ends (Figure 10) and lead to the final ED via a two-step mechanism. For the fast pathway, the kinetic and equilibrium constants for IC formation are very close to those of the Gag-promoted cTAR/dTAR annealing, suggesting the formation of the same IC with the two proteins. The main difference between the two proteins is their efficiency in converting the IC into the ED. As shown by the four-fold higher *k*_f_ value observed with RPL7 as compared to Gag, the former appears as a more efficient promoter of IC conversion, suggesting that the RPL7 binding site encompasses residues of the middle of cTAR and dTAR stems involved in the conversion of the IC into the ED. Therefore, from the similarities with the Gag-promoted annealing reaction, the fast pathway likely corresponds to a ‘zipper’ pathway, in which spatially close RPL7-bound cTAR and dTAR species are simultaneously melted at their stem ends, so that they can anneal their both strands. Since RPL7 does not exhibit any destabilization activity (Figure 5A), the melting of the stem ends results from their thermal fraying (58,64). Since the IC is substantially less stable in the slow pathway, we hypothesized that in this pathway, one strand of the melted stem of a RPL7-bound ODN may invade the closed stem of a spatially close RPL7-bound complementary species. Therefore, only ∼4 bps can form (Figure 10) instead of 7 bps for the IC in the fast ‘zipper’ pathway. This lower number of base pairs is also consistent with the 6-fold slower conversion of the IC into the ED as compared to the ‘zipper’ mechanism. Our model is further substantiated by the disappearance of the slow pathway with dTAR1,2, likely due to the high stability of its stem that hinders the RPL7-driven invasion by the melted stem end of cTAR. Moreover, both the increased stability of the cTAR/dTAR1,2 IC and its slow conversion rate are in line with the additional stability provided by the closure of the upper bulge in dTAR1,2. Interestingly, the two pathways are preserved with the dTAR-switch mutant. For the fast pathway, the fast dissociation of the IC and its fast conversion into ED suggest that RPL7 is endowed with efficient matchmaker properties that facilitate the search for the most stable base pair combination between the two strands. For the slow pathway, a highly stable IC that can only be converted at slow rate into ED is formed. The nature of this stable IC is unknown.

For the Gag–RPL7 complex, the promotion of cTAR/dTAR annealing is clearly more pronounced than for the isolated proteins, suggesting that the two proteins bind simultaneously to cTAR and do not compete for a single binding site. The effect of the complex on the cTAR/dTAR annealing mechanism (Figure 9) is clearly dominated by Gag, as can be seen by the preservation of the single two-step pathway and the number of base pairs that need to be melted to initiate the IC formation. The dominant effect of Gag is also highlighted by the value of the IC conversion rate, which is close to that of Gag alone. Thus, Gag alone or in complex with RPL7 likely binds to the same binding site. The main effect of RPL7 in the complex is to stimulate the rate of IC formation, likely by further neutralizing the negative charges of the DNA reactants, and thus promoting the association of the protein/DNA complexes. The RPL7 protein also increases the dissociation rate of the IC, so that the overall stability of the IC remains the same as for Gag alone. RPL7 can thus be perceived as a helper for the NAC activity of Gag that facilitates the nucleation of the IC and at the same time prevents its trapping in a too stable form. In contrast, RPL7 only increases the conversion rate *k_f_*by 2-fold in respect to Gag alone. The strong differences between the Gag–RPL7 complex and RPL7 alone suggest that RPL7 adopts a different binding mode to the DNA reactants when it is associated to Gag. Using the dTAR-TL mutant, it was observed that as for Gag, the dTAR loop plays a modest role in the Gag–RPL7-promoted annealing reaction, by facilitating the conversion step. The most remarkable contribution of RPL7 in complex with Gag was observed with dTAR1,2 where it almost fully counteracts the effects of the increased stability of dTAR1,2. In comparison to Gag alone, the Gag–RPL7 complex increases the values of all kinetic parameters by a factor of 7 to 40 (Table 3). Thus, RPL7 acts as a helper which promotes the NAC activity of Gag, especially for stable sequences. This was clearly confirmed by the annealing of (+)PBS RNA to the highly stable tRNA^Lys^_3_ where RPL7 boosted the NAC activity of Gag, with an efficiency even higher to that of IP6, a previously reported co-factor of Gag (10). The values of all kinetic rate constants for the Gag–RPL7-promoted annealing of cTAR with dTAR-switch are about 5 to 10-fold slower than for the corresponding annealing with the native dTAR, suggesting that transient interactions between properly oriented reacting NA species are involved in the Gag–RPL7-promoted formation and dissociation of cTAR/dTAR IC, and its conversion into the ED.

In conclusion, our data clearly confirm that Gag acts as a NAC, endowed with NA destabilization and annealing properties (42–43,76). This activity is likely dominated by its NC domain, as Gag was found to promote cTAR/dTAR annealing through the stem ends with the same mechanism than the mature NCp7 (50,77). The NAC activity of Gag was found to strongly depend on the stem stability and notably on the presence of the two bulges which destabilize the lower part of the cTAR stem (64,73). In addition, the close proximity and proper orientation of the complementary NA sequences in the IC are thought to play an important role in its conversion into the ED. In contrast, the loops play only a modest role, being involved in the IC conversion into ED (73). In opposition to Gag, RPL7 does not destabilize cTAR but efficiently promotes its annealing with dTAR through a two-pathway mechanism with an efficient conversion of the ICs into the ED. The properties of RPL7 are similar to those already reported for a HCV core peptide (51) and thus, are typical of a NA annealer (78,79). In association with Gag, RPL7 was found to act as a cofactor that stimulates the NAC activity of Gag. This effect is particularly pronounced with stable NA sequences, in full line with our data on tRNA^Lys^_3_/PBS (Figure 8) and our previous paper (48), where we showed that the Gag–RPL7 complex but not the isolated proteins efficiently promote the annealing of cTAR with TAR RNA which is more stable than dTAR by 18 kcal/mol, as calculated with mFold. While most of our conclusions were obtained with a molar ratio of protein to NAs of 1:1, the data on tRNA^Lys^_3_/PBS RNA indicated that the conclusions with 2:1 and 1:1 molar ratios were similar (Table 5). Considering that in the initial steps of viral assembly, the genomic RNA interacts mainly with Gag monomers/dimers (80–82) and traffics to the plasma membrane with relatively few Gag proteins (83), the low molar ratios used in our study can be considered relevant for locally describing the NAC activity of Gag on the genomic RNA *in vivo*. Therefore, the ability of RPL7 to boost the NAC properties of Gag on stable sequences is thought to help Gag to overcome roadblocks in the viral assembly process.

## Supplementary Material

gkaa659_Supplemental_File
