# Supplementary material for: The nucleic acid chaperone activity of the HIV-1 Gag polyprotein is boosted by its cellular partner RPL7: a kinetic study
Source: Nucleic Acids Res. 2020 Aug 14;48(16):9218–34. doi: 10.1093/nar/gkaa659 (PMC7498347; doi:10.1093/nar/gkaa659)
Supplement: gkaa659_Supplemental_File [file gkaa659_supplemental_file.pdf]

Supporting information

The nucleic acid chaperone activity of the HIV-1 Gag polyprotein is boosted by its cellular partner RPL7: a kinetic study

Karnib Hassan[1,#], Nadeem Muhammad Faisal[1,#], Nicolas Humbert[1, #], Sharma Kamal Kant[1], Grytsyk Natalia [1], Tisné Carine [2], Boutant Emmanuel[1], Lequeu Thiebault[1], Real Eleonore[1], Boudier Christian[1], de Rocquigny Hugues [3*] and Mély Yves[1*]

[1]Laboratory of Bioimaging and Pathologies (LBP), UMR 7021, Faculty of pharmacy, University of Strasbourg, 67400 Illkirch, France

[2]Expression génétique microbienne, UMR 8261, CNRS, Université de Paris, Institut de biologie physico-chimique, 13 rue Pierre et Marie Curie, 75005, Paris, France.

[3]Inserm – U1259 Morphogenesis and Antigenicity of HIV and Hepatitis Viruses (MAVIVH), 10 boulevard Tonnellé, BP 3223, 37032 Tours Cedex 1, France

* *yves.mely@unistra.fr ; hderocquigny@univ-tours.fr*

Check of protein purity

SDS-PAGE & western-blot

To check Gag purity, 10 µg of the final purified Gag protein was loaded on a 10% SDS PAGE gel. The Color Prestained Protein Standard (P7712; New England Biolabs) was used as a molecular weight ladder on the same gel. The gel was revealed by incubation with Coomassie blue (GEN-QC-STAIN; Generon) for at least 2 hours at room temperature. The plot profile was obtained by using ImageJ, without any data pre-treatment.

The western-blot transfer was performed using a polyvinylidene difluoride (PVDF) membrane from an induction test input. The blot was blocked with a 3% of Non-Fat Dry Milk Solution (Sigma) diluted in Tris-buffered saline-Tween 20 (TBST). The membrane is then incubated 1 h with the anti-His/HRP conjugated antibody (631210; Clonetech). The signal is revealed with the ECL solution (Clarity; 170-5061; Biorad) and recorded with the Image Quant LAS4000 (GE Healthcare).

Size exclusion chromatography

Size exclusion chromatography was performed on a Superdex 200 resin packed in a XK16/60 column connected to an AKTA protein purification system (GE Healthcare) controlled by the Unicorn 5.3 software. The column was washed with 3 column volumes (CV) of MilliQ water then 3 CV of 50 mM Tris pH=8, 1 M NaCl buffer with a flow rate of 0.5 mL/min. A 50 µL sample of Gag Protein was loaded on the column by using a 100 µL loop and the total run lasted for 1,5 CV.

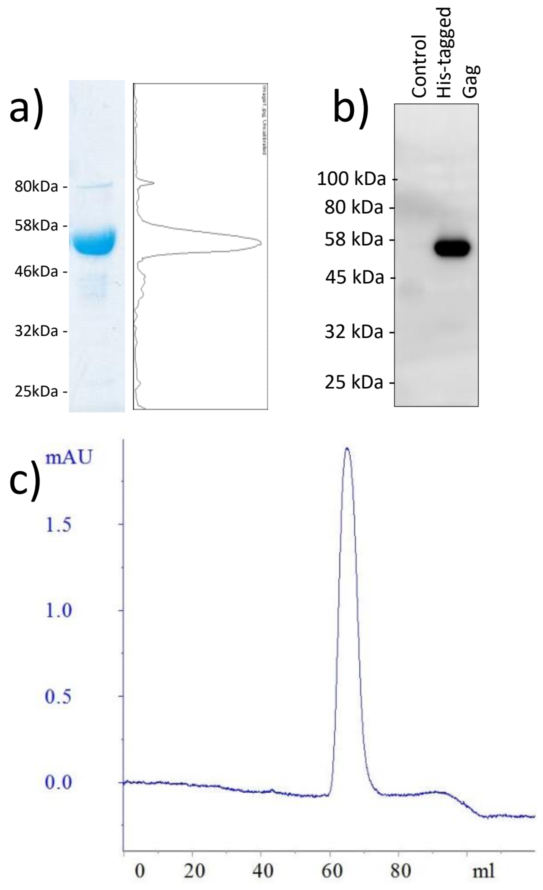

Figure S1: Purity check of the prepared Gag protein. a) SDS-PAGE gel electrophoresis with Coomassie blue staining of 10 µg purified Gag protein. Gag appears as a single broad band at 57 kDa. The plot profile on the right clearly shows that the Gag band is highly predominant (> 90%). b) Western-blot of the His-tagged Gag protein before the final cleavage step. The protein is revealed with an anti-His-HRP antibody (1/5000). This blot confirms the size (57 kDa) and specific expression of the Gag protein. The control was performed on the cleared lysate of non-induced bacteria. c) Control chromatogram with 50 µL of the final purified Gag product. The elution profile shows a single symmetric peak that confirms the high purity of the protein and the absence of protein aggregation.

FCS data

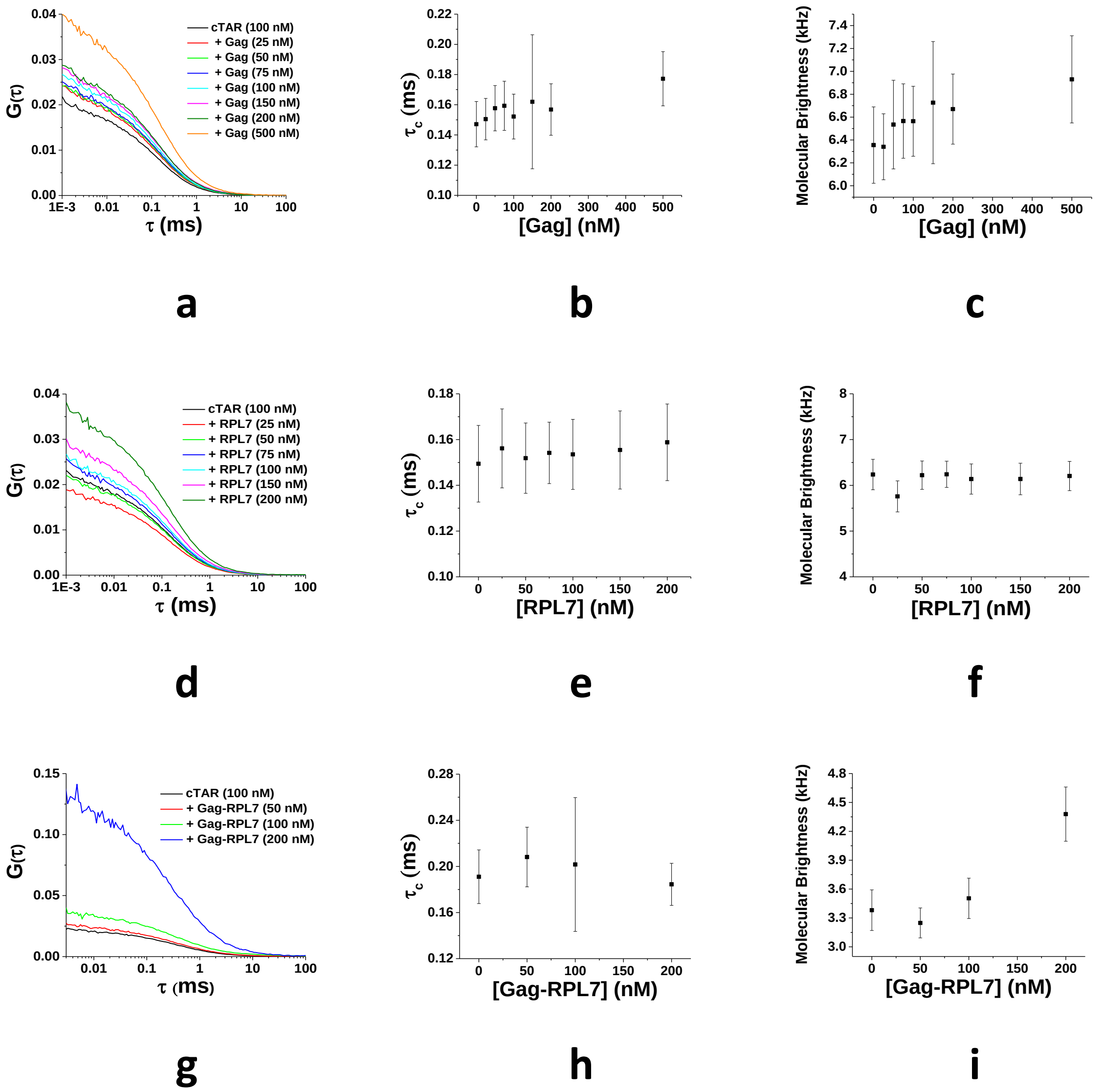

Figure S2: Fluorescence correlation spectroscopy analysis of the interaction of cTAR with Gag (a-c), RPL7 (d- f) and the Gag-RPL7 complex (g-i). The autocorrelation curves (a, d, g) were recorded with 100 nM TMR-5'-cTAR and increasing concentrations of Gag, RPL7 and Gag-RPL7. The correlation times were extracted from the fits of the autocorrelation curves with the free 3D diffusion equation 1 (b, e, h). The limited increase in the correlation time observed with all proteins could be rationalized by the non-spherical shape of cTAR and the cubic square root dependence of the diffusion time on the molecular weight of the diffusing species. The brightness (c, f, i) of the diffusing species is obtained by dividing the average fluorescence intensity in the focal volume by the average number N of fluorescent species in this volume. The brightness per diffusing species (Fig S1 c, f and i) was found to be almost constant for Gag

and RPL7 (up to 200 nM), and Gag-RPL7 (up to 100 nM), confirming that the diffusing species contain a single TMR-5'-cTAR molecule and indicating that the quenching of the TMR fluorescence in the complexes of TMR-5'-cTAR with the proteins is marginal. From 85% to 100% of the 50 to 60 individual autocorrelation curves were considered for the fit of the FCS data, with the exception of 200 nM Gag-RPL7, where the percentage dropped to 50 %.

Binding of Gag, RPL7 and Gag-RPL7 to cTAR

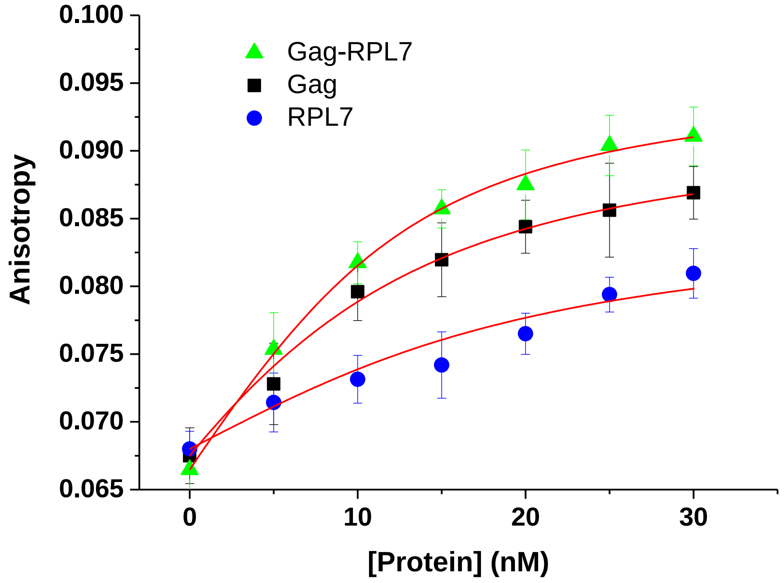

Figure S3. Fluorescence anisotropy titration of 10 nM Fl-5'-cTAR with increasing concentrations of Gag (black squares), RPL7 (blue circles) and their complex Gag-RPL7 (green triangles). The fits of the data points with equation 2 are shown as red lines. Excitation and emission wavelengths were 480 nm and 520 nm, respectively. Measurements were performed at 20°C in 50 mM Tris, 150 mM NaCl, 1 mM $MgCl_2$, 1 mM DTT (pH 7.4).

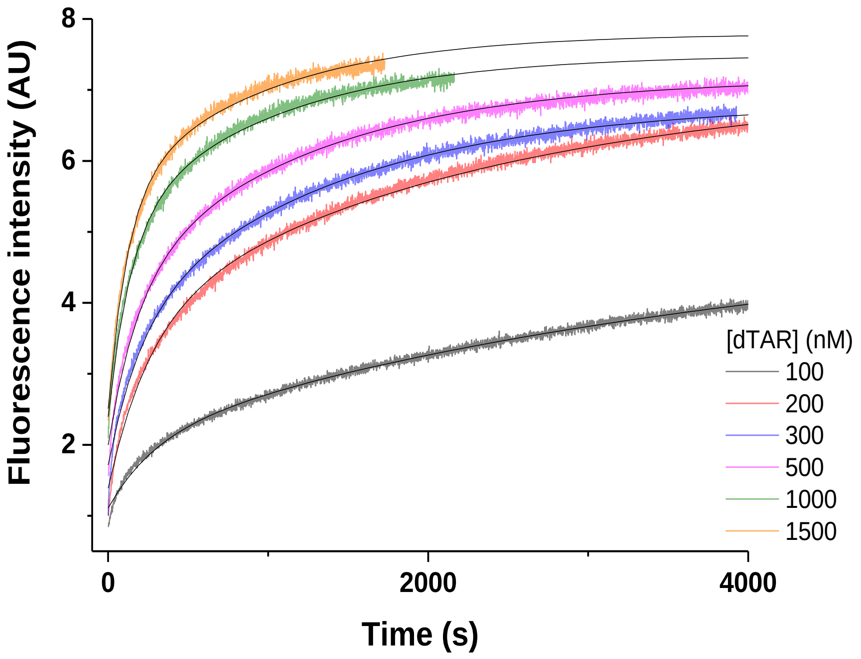

Figure S4: Progress curves for the Gag-promoted hybridization of Rh6G-5'-cTAR-3′-Fl to dTAR under pseudo-first order conditions. 10 nM doubly labeled cTAR was reacted with 100, 200, 300, 500, 1000 and 1500 nM dTAR in the presence of Gag added at a protein/ODN ratio of 1. The black smooth curves were generated by analyzing the set of kinetic traces by the Dynafit software in agreement with Scheme 1 and the best estimates of k_{ass}, k_{diss} and K_a* (Table 1). All curves were recorded using an excitation and emission wavelength of 520 and 555 nm, respectively. Buffer was 50 mM Tris-HCl (pH 7.4), 150 mM NaCl, 1 mM MgCl2 and 1 mM DTT at 20°C.

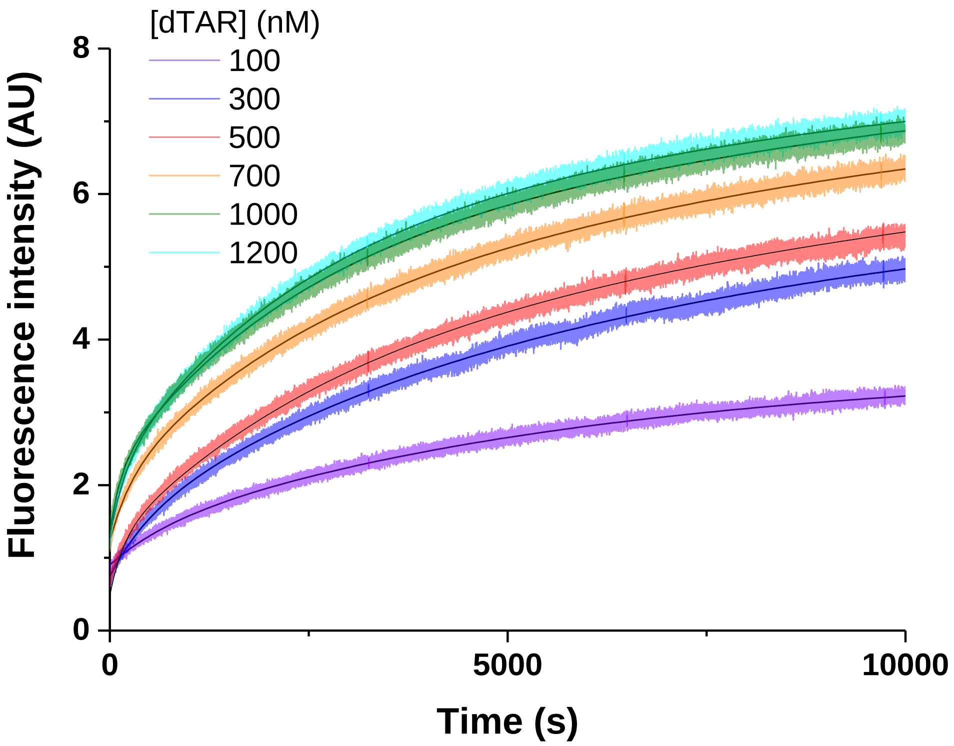

Figure S5: Progress curves for the Gag-promoted reaction of 10 nM doubly labelled cTAR with increasing concentrations of dTAR-switch. Gag was added at a protein : ODN molar ratio of 1:1. The black curves were generated by fitting the set of experimental traces by the Dynafit software using the Scheme 3 model and the best estimates of the kinetic parameters listed in Table 1. The dTAR-switch concentrations were 100 nM, 300 nM, 500 nM, 700 nM, 1000 nM, and 1200 nM. The experimental conditions are identical to those in **Figure S4**.
